# The Combination of Shaking and Yellow-Light Withering Promote the Volatile Aroma Components and the Aroma Quality of Black Tea

**DOI:** 10.3390/foods14050758

**Published:** 2025-02-23

**Authors:** Zeyi Ai, Shuangming Hu, Lingfei Ji, Bing Mu, Yiyang Yang

**Affiliations:** Jiangsu Key Laboratory for Horticultural Crop Genetic Improvement, Institute of Leisure Agriculture, Jiangsu Academy of Agricultural Sciences, Nanjing 210014, China; aizeyi@jaas.ac.cn (Z.A.); lingfeiji@jaas.ac.cn (L.J.);

**Keywords:** black tea, shaking combinations, yellow-light withering, volatile substance, aroma improvement

## Abstract

The application of shaking during the withering process has been shown to significantly enhance the floral aroma of black tea. However, prior to this study, there was limited research on the effects of shaking combined with other withering treatments on the aroma components of black tea. In this study, the aroma attributes of black teas processed with shaking combinations of yellow-light or high-temperature withering (YLS, HTS, and HYS) were evaluated through sensory evaluation, and the volatile composition and identification of key aroma compounds in black teas and in-process withered tea leaves were analyzed by gas chromatography–mass spectrometry (GC-MS). The results indicated that black teas subjected to different shaking combinations exhibited a distinct floral aroma with higher volatile compound content, with the YLS treatment showing the most significant aroma improvement. Eight volatile compounds with relative odor activity values (rOAV) > 1 were identified from 53 differential volatile compounds in black tea under different shaking combinations as the most important contributors to aroma quality. Linalool, trans-β-Ionone, α-cedrene, and nonanal were identified as key floral volatiles with high rOAVs. Their concentrations in YLS were notably higher compared to those in S, both in withered tea leaves (with the exception of trans-β-Ionone) and in the final dry black teas, suggesting that there may be a synergistic effect between the yellow-light withering and the shaking process in enhancing these key floral compounds. Overall, this study found that shaking combined with yellow-light withering can improve the aroma composition and quality of black tea, providing a theoretical basis and practical guidance for the production and optimization of high-aroma black tea.

## 1. Introduction

Black tea, renowned globally for its distinct, diverse, and rich aroma, is the most widely produced and consumed tea type worldwide [1]. The aroma of black tea is characterized by sweet, floral, honey, and caramel-like notes [2,3]. Aroma performance is constituted by the mixture of multiple volatile flavor compounds (VFCs) [4,5], making the study of VFCs a focal point in tea research. To date, over 600 aroma components have been identified in black tea, primarily consisting of aldehydes, alcohols, esters, alkanes, and ketones [6]. Among these, geraniol (rose floral), trans-β-ionone (floral), methyl salicylate (floral), (Z/E)-linalool oxides (floral), linalool (sweet floral), phenylacetaldehyde (honey), and β-damascenone (sweet, honey-like) amongst others are recognized as the predominant aroma-active compounds [7,8], which are primarily generated during tea processing.

The processing of black tea involves several stages: withering, rolling/rolling and cutting, fermentation, and drying. Withering, the initial step, plays a crucial role in shaping the overall aroma quality of black tea [9,10]. Appropriate withering techniques can promote the favorable transformation of volatile aroma compounds in fresh tea leaves, thereby enhancing the tea’s aroma quality [11]. With continuous innovation in withering technology, many black-tea-producing regions in China have incorporated shaking into the withering process to enrich flavor, resulting in what is known as shaken black tea (SBT) [12]. Shaking involves rotating withered leaves using a water sieve or shaker machine. Studies have shown that shaking facilitates friction-induced damage along the leaf edges, stimulating enzyme activities such as oxidase, hydrolase, and reactive oxygen species metabolism enzymes [13], thereby promoting the synthesis of floral compounds like indole, methyl jasmonate, and β-ionone, and significantly enhancing the floral flavor of black tea [14,15].

Additionally, previous research demonstrated that yellow, orange, and red-light withering can significantly improve the aroma and taste quality of black tea, with yellow light particularly effective in increasing the content of VFCs such as linalool and geraniol [16]. Numerous studies also highlighted the role of light quality in tea spreading and withering processes. For instance, yellow light combined with low-temperature treatment markedly enhanced the synthesis and accumulation of methyl salicylate and indole during green tea spreading [17], while red-light withering primarily influenced the accumulation of terpene volatile components in the later stages of black tea withering by upregulating key synthase gene expression [18]. Furthermore, warm-air withering is commonly used in black tea production to accelerate water loss and shorten withering time [19], though there is limited research on the effects of high-temperature withering on black tea aroma components.

While existing research has primarily focused on the impact of individual technologies on black tea quality, there is a lack of systematic investigation into the combined effects of multiple withering technologies, particularly on aroma quality. The influence of different combinations of yellow light, high temperature, and shaking on the metabolism of volatile compounds in black tea remains unclear. Therefore, this study explores the effects of different shaking combinations of yellow-light or high-temperature withering on the volatile aroma compounds of black tea, aiming to provide theoretical and practical insights for the application of various withering techniques in black tea production and to lay the groundwork for optimizing processing technologies for high-quality black tea.

## 2. Materials and Methods

### 2.1. Reagents and Equipment

Ethyl decanoate (≥99% purity) was purchased from Macklin Biochemical Technology Co., Ltd. (Shanghai, China). A C3-C25 N-alkane mixture was obtained from o2si smart solutions International LLC (North Charleston, SC, USA). The TSQ 8000 EVO gas chromatograph-mass spectrometer (GC-MS) was purchased from Thermo Fisher Scientific, Inc. (Waltham, MA, USA). Supelco SPME fiber (PDMS/DVB 65 µm) was purchased from Merck KGaA (Darmstadt, Germany). The LCWD-5 withering machine, 6CWL-90 shaker machine, and 6CFJ-0.7 fermentation machine were obtained from Fujian Jiayou Tea Machinery Intelligent Technology Co., Ltd. (Anxi, China). Yellow light-emitting diode (LED) lamps (cold light source, 585–590 nm, peak wavelength 587.9 nm) and the LI-250A light meter were purchased from Zhongshan Light of Sea Emperor Co., Ltd. (Zhongshan, China) and LI-COR Biosciences Co., Ltd. (Lincoln, NE, USA).

### 2.2. Tea Sample Processing

**Figure 1:**
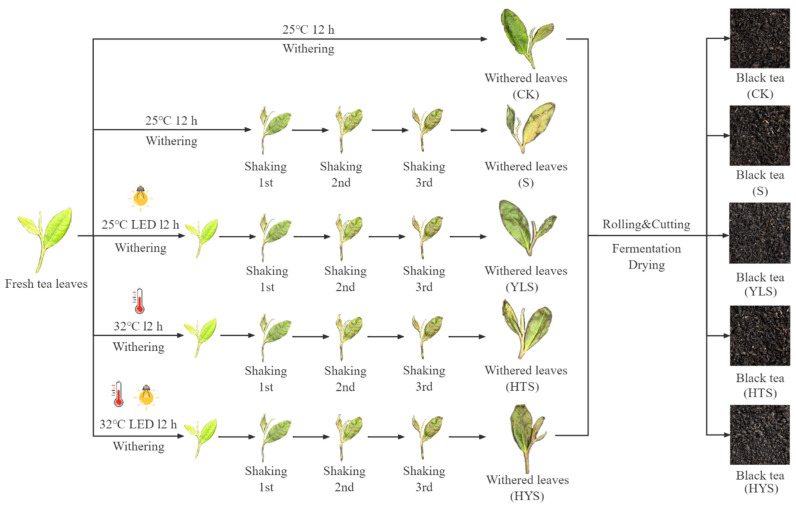
The experimental processing flowchart of black tea.

Fresh tea leaves with one bud and two leaves were harvested from the Lj43 cultivar (*Camellia sinensis var. sinensis cv. Lj43*) in modern tea plantations in Pukou County at 32.101216° N, 118.657506° E (Nanjing City, Jiangsu Province, China) in July 2023. The tea shoots were subjected to five different withering treatments: indoor natural withering (CK), shaking (S), yellow light plus shaking (YLS), high temperature plus shaking (HTS), and high temperature with yellow light plus shaking (HYS). For the CK treatment, tea leaves were withered in a withering machine (LCWD-5) for 12 h at 25 °C and 60% relative humidity (RH) with natural light intensity below 200 lux. For the S treatment, after 12 h of indoor natural withering, the tea leaves were shaken three times using a shaker machine (6CWY-90) for 10 min each, with 15 min intervals between shakes. For the other treatments, tea leaves were withered under yellow-light irradiation (YLS, 25 °C, 60% RH, 844–864 lux), at a high room temperature (HTS, 32 °C, 60% RH, <200 lux), or at a high room temperature with yellow-light irradiation (HYS, 32 °C, 60% RH, 844–864 lux) before shaking. The shaking parameters were consistent with those of the S treatment. During the 12 h withering process, the leaves were stirred every hour to ensure uniform light exposure. After five different treatments, the tea leaves underwent further withering by fan in withering machine (LCWD-5) until their moisture content decreased to 60.0 ± 1.0%, respectively. Subsequently, half of the withered leaves were frozen in liquid nitrogen and stored at −80 °C for subsequent experiments, while the other half were processed into black tea through rolling and cutting (2 times), fermentation (30 °C, 90% RH for 3 h), and drying (110 °C for 30 min, then 80 °C until the moisture content reached approximately 6%). Each treatment was repeated three times, and the experimental processing flowchart is shown in Figure 1.

### 2.3. Sensory Evaluation

Black tea samples were prepared from tea leaves subjected to the five postharvest treatments (CK, S, YLS, HTS, and HYS). Sensory evaluation was conducted by five professional tea tasters with Senior Tea Assessor qualifications (China National Professional Qualification Level 3). Following the Chinese Tea Evaluation Standard Procedure (GB/T 23776-2018), the appearance of dry tea was evaluated first. After brewing 3.0 g of dry tea with 150 mL of boiling water for 5 min, the brew color, aroma, taste, and infused leaves were assessed. Scores were based on a 100-point scale, with appearance accounting for 20%, infusion color for 10%, aroma for 30%, taste for 30%, and infused leaves for 10% [20]. Sensory assessments were conducted blindly and independently by the five panelists.

### 2.4. Extraction and Analysis of Volatile Compounds

Volatile compounds were extracted using the headspace solid-phase microextraction (HS-SPME) method. For withered tea leaves, 2.000 g of freeze-dried powder was weighed into a 20 mL headspace bottle, and 100 µL of ethyl decanoate (1 µL/L) was added as an internal standard. The bottle was sealed, and an SPME fiber (65 µm PDMS/DVB) was inserted and heated in a 60 °C water bath for 60 min to adsorb the aroma components. For black tea, 2.000 g of crushed tea was mixed with 10 mL of boiling distilled water and 100 µL of ethyl decanoate (4 µL/L, internal standard) and extracted using a 50/30 μm CAR/DVB/PDMS SPME fiber for 60 min in a 60 °C water bath [18]. Each sample was analyzed in triplicate.

Volatile compounds were analyzed using a GC-MS. After extraction, the fiber was immediately inserted into the GC injector for thermal desorption at 250 °C for 5 min. The analysis was performed under the following conditions: chromatographic column: TG-5MS (30 m × 0.25 mm × 0.25 µm, Thermo Fisher Scientific, USA); injection mode: splitless; carrier gas: high-purity helium (purity ≥ 99.99%); column flow rate: 1.2 mL/min; inlet temperature: 250 °C; heating program: initial temperature of 35 °C held for 2 min, increased to 180 °C at 4 °C/min and held for 2 min, then increased to 280 °C at 15 °C/min and held for 5 min; ion source (EI) electron energy: 70 eV; ion source temperature: 300 °C; mass scanning range (m/z):35–400 amu. Volatiles were identified by matching the National Institute of Standards and Technology mass spectral database (NIST 17, USA) and retention index (RI) [21]. The RI for each compound was calculated using the linear formula for C3-C25 N-alkanes. Compounds were further screened based on the retention index difference compared to the NIST database.

### 2.5. Quantification and Relative Odor Activity Value (rOAV) Calculation

Xcalibur software (Thermo Fisher Scientific, Waltham, MA, USA) was used for GC-MS data analysis, including peak extraction, baseline filtering, calibration, peak alignment, deconvolution, and peak area integration. Volatile compounds were semi-quantified based on the peak areas of the internal standard. rOAVs were calculated by dividing the content of each volatile compound by its odor threshold in water [22]. Odor thresholds (OTs) in water were based on the literature [5,15,23,24].

### 2.6. Data Processing Method and Statistical Analysis

All data are presented as means ± standard deviation (SD). Significant differences were determined using one-way analysis of variance (ANOVA) in SPSS 20.0 (SPSS, Inc., Chicago, IL, USA) based on Duncan’s multiple-range test (*p* < 0.05). Partial least squares discriminant analysis (PLS-DA) was performed using MetaboAnalyst 3.0 (https://www.metaboanalyst.ca/ accessed on 18 October 2023). Figures were generated using Processon (https://www.processon.com/ accessed on 23 August 2024), GraphPad Prism 9 (GraphPad Software company, Boston, MA, USA) and Adobe Illustrator CC 2019 (Adobe Systems Incorporated, San Jose, CA, USA).

## 3. Results and Discussion

### 3.1. Sensory Quality of Black Tea Under Different Shaking Combinations

Sensory evaluation revealed that shaking and shaking-involved combinations significantly influenced the aroma, taste, and infusion color of black tea, with less impact on appearance and infused leaf quality. As shown in Table 1, the brightness of infusion color under high-temperature withering combinations (HTS and HYS) were both lower than that of CK, suggesting that high-temperature withering may negatively affect infusion color. Compared to CK, all shaking combinations positively influenced black tea taste, except for HYS, which exhibited a less mellow taste. Regarding aroma quality, all shaking combinations resulted in a distinct floral aroma alongside a sweet aroma. In contrast to a slightly floral and sweet aroma of HTS and HYS, the floral and sweet aroma of YLS and S was stronger and more persistent. It indicated that shaking is crucial for forming the floral aroma of black tea, while additional withering treatments (yellow light or high temperature) can influence aroma intensity and persistence instead of the aroma type. The combination of yellow-light withering and shaking (YLS) achieved the highest aroma score, suggesting that yellow-light withering may further enhance the aroma quality of shaken black tea.

### 3.2. Volatile Compound Analysis of Black Tea Under Different Shaking Combinations

#### 3.2.1. Qualitative and Quantitative Analysis of Volatile Compounds

**Figure 2:**
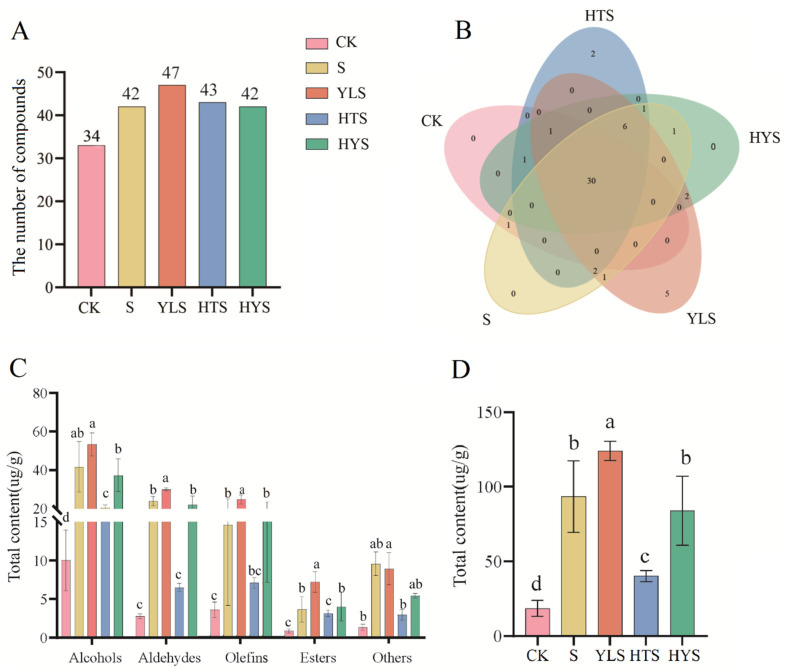
The distribution of volatile compounds in black tea under different withering combinations. (**A**) The number of volatile compounds identified in each withering treatment. (**B**) Venn diagram showing the overlap and unique volatile compounds among the five withering treatments. The numbers in the diagram represent the count of volatile compounds in each overlapping or unique region. (**C**) Total volatile contents of the five groups categorized by chemical class. (**D**) Total contents of all the volatiles. The total content of volatiles was presented as mean ± SD (n = 3). Different lowercase letters above the bars in the same volatile category indicate significant differences at *p* < 0.05. Treatments: CK represents indoor natural withering, S represents shaking, YLS represents yellow light plus shaking, HTS represents high temperature plus shaking, and HYS represents high temperature with yellow light plus shaking.

A total of 53 volatile components were identified across different black tea samples (Table S1), including fourteen alcohols, twelve olefins, eleven esters, nine aldehydes, two ketones, one ether, one organosulfur compound, one aromatic compound, one heterocyclic compound, and one acid. The addition of shaking or shaking combinations during withering increased the number of volatile substances in black tea, with the YLS treatment group showing the highest number of volatile compounds (Figure 2A). Venn diagram analysis revealed 30 common volatile compounds across all samples, with six overlapping volatiles in four shaking-involved treatments, including benzyl alcohol, cis-3-hexenyl isovalerate, cedryl acetate, β-copaene, D-limonene, and 2-pentyl-furan (Figure 2B). Five components, such as β-guaiene, α-alaskene, 10-undecenyl-3-phenylpropanoate, 2-ethyl-1-hexanol, and geranyl vinyl ether, were unique to YLS, while geranyl isovalerate and trans-2-hexenoic acid were unique to HTS. No unique substances were detected in S and HYS. These findings suggest that shaking increases the variety of volatile compounds, and additional withering treatments (YLS and HTS) can further enhance this effect, thus changing the volatile composition of black tea.

Quantitative analysis showed that different shaking combinations improved the content of various volatile categories (Figure 2C). YLS had the highest content of alcohols, aldehydes, olefins, esters, and other compounds, leading to the highest level in total volatile content (Figure 2D). Compared to CK, S and HYS also promoted the accumulation of these categories, while HTS only significantly increased alcohol content. Major alcohols such as linalool, cedrol, and trans-linalool oxide (furanoid) increased significantly after shaking combinations, contributing to the overall increase in alcohol content. Similarly, S, YLS, and HYS increased the content of main aldehydes (e.g., 3-methylbutyraldehyde), olefins (e.g., β-cedrene and α-cedrene), esters (e.g., α-terpinyl acetate), and other compounds (e.g., dimethyl sulfide). The total content of these categories exhibited the following order: YLS > S ≈ HYS > HTS > CK, indicating that YLS is most beneficial for volatile compound formation in black tea, thereby enhancing aroma quality, which was consistent with the aroma scores of sensory evaluations.

#### 3.2.2. PLS-DA Analysis of Volatile Compounds

**Figure 3:**
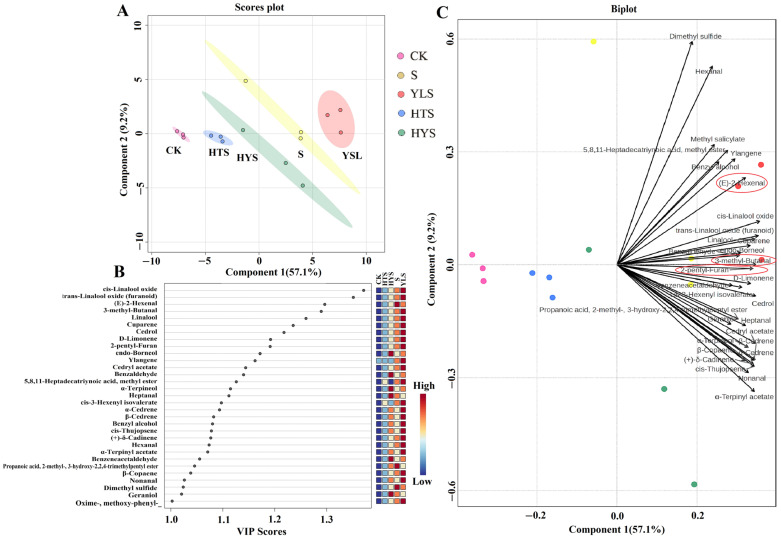
PLS-DA analysis of main components in black teas under different shaking combinations. (**A**) Score plot showing the distribution of samples based on the first two principal components. Each point represents a sample, and the ellipses indicate confidence intervals for each group. (**B**) VIP (Variable Importance in Projection) plot displaying the key volatile compounds (VIP scores > 1.0) contributing to the separation of groups with. (**C**) Biplot illustrating the relationship between samples (points) and volatile compounds (vectors) in the PLS-DA model. Treatments: CK represents indoor natural withering, S represents shaking, YLS represents yellow light plus shaking, HTS represents high temperature plus shaking, and HYS represents high temperature with yellow light plus shaking.

The partial least squares discriminant analysis (PLS-DA) was shown in Figure 3. The model parameters (R2X(cum) = 0.934, R2Y(cum) = 0.986, Q2(cum) = 0.925) indicated high credibility and predictive ability. Principal component 1 (57.1%) and principal component 2 (9.2%) effectively separated and predicted black tea samples under different shaking combinations (Figure 3A). Apparently, the tea samples were clearly classified into five groups, with CK on the far left and YLS on the far right showed the largest difference, which validated the results of sensory evaluation. HTS is just next to CK, and they are both on the left side of the horizontal axis. HYS approaches the origin and S is positioned between HYS and YLS. These results confirm that different shaking combinations significantly affect the composition and content of volatile components in black tea.

According to the value of variable importance factor (VIP > 1, *p* < 0.05), thirty-one compounds were identified as key components differentiating black teas under different shaking combinations, with their contents significantly promoted in YLS (Figure 3B). The biplot (Figure 3C) further illustrated the contributions of these volatiles to classification, with most of the key differentiating compounds are in the right quadrant near S and YLS. It was noted that 3-methyl-butanal, (E)-2-hexenal and 2-pentyl-furan were closer to YLS, indicating their significant influence on aroma differentiation.

#### 3.2.3. Key Aroma Compounds of Black Teas

The relative odor activity values (rOAVs) were calculated to evaluate the contribution of specific volatiles to black tea aroma (Table S2). Eight compounds (rOAV > 1) were identified as the most important contributors to black tea aroma under different shaking combinations (Table 2), including linalool, cedrol, 3-methyl-butanal, trans-β-ionone, dimethyl sulfide, benzeneacetaldehyde, α-cedrene, and nonanal. Among these, linalool, cedrol, 3-methyl-butanal, and trans-β-ionone had average rOAV values > 10, indicating their substantial contributions to black tea aroma.

Compared to CK, the rOAV values of key aroma components increased significantly after shaking combinations, particularly in YLS, which had the highest rOAVs for all aroma-active compounds, suggesting superior aroma quality, further corroborated the results of sensory evaluation. Trans-β-ionone in HYS (78.50 ± 32.76) and dimethyl sulfide in S (12.35 ± 4.39) also showed high rOAVs. Referring to the previous literature for the aroma description (Table 2), linalool, trans-β-ionone, α-cedrene, and nonanal were identified as key floral aroma substances, with their highest rOAV values in YLS, indicating that the increase in floral aroma compounds is crucial for aroma quality improvement under YLS treatment.

### 3.3. Volatile Compounds Analysis of Withered Leaves under Different Shaking Combinations

#### 3.3.1. Overall Analysis of Volatile Compounds

**Figure 4:**
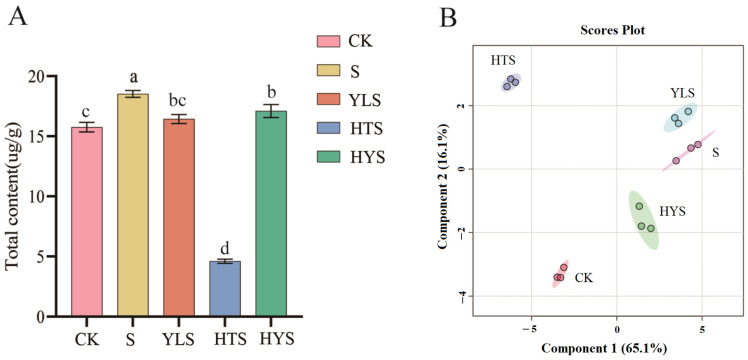
Effect of different shaking combinations on volatile components in withered leaves. (**A**) The total content of volatile components in withered leaves under different shaking combinations (mean ± SD, n = 3). Different lowercase letters above the bars indicate significant differences at *p* < 0.05. (**B**) Score plot of PLS-DA (partial least squares discriminant analysis) showing the distribution of samples based on the first two principal components. Each point represents a sample, and the ellipses indicate confidence intervals for each group. Treatments: CK represents indoor natural withering, S represents shaking, YLS represents yellow light plus shaking, HTS represents high temperature plus shaking, and HYS represents high temperature with yellow light plus shaking.

In total, 52 volatile components were obtained from in-process withered leaves (Table S3). The total volatile content in withered leaves increased significantly after S and HYS treatments compared to CK, while YLS showed a non-significant increase (Figure 4A). HTS exhibited a decrease in volatile content, suggesting that high-temperature withering may hinder volatile compound formation in withered leaves. Moreover, PLS-DA analysis of withered leaves (Figure 4B) showed distinct clustering of samples under different treatments, with S and YLS in the first quadrant, HTS in the second, CK in the third, and HYS in the fourth, indicating significant differences in the volatile composition of withered tea leaves.

#### 3.3.2. Analysis of Key Aroma Components

**Figure 5:**
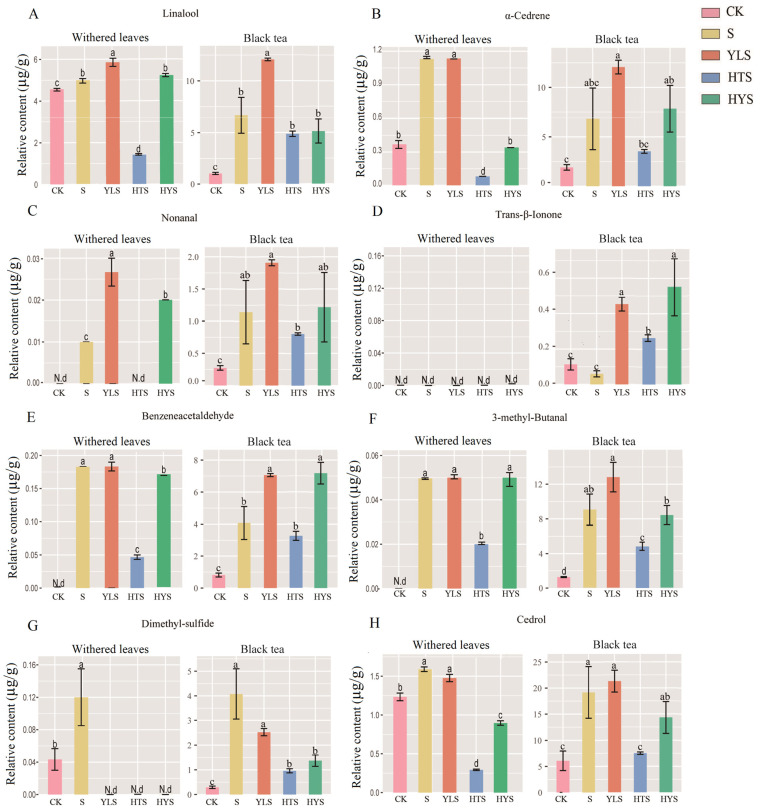
The relative contents of key aroma components in withered leaves and black teas: (**A**) linalool, (**B**) α-cedrene, (**C**) nonanal, (**D**) trans-β-ionone, (**E**) benzeneacetaldehyde, (**F**) 3-methyl-butanal, (**G**) dimethyl sulfide, and (**H**) cedrol. The relative contents were presented as mean ± SD (n = 3). Different lowercase letters above the bars in the same volatile category indicate significant differences at *p* < 0.05. N.d denotes that the component was not detected in the sample. Treatments: CK represents indoor natural withering, S represents shaking, YLS represents yellow light plus shaking, HTS represents high temperature plus shaking, and HYS represents high temperature with yellow light plus shaking.

The key aroma components of withered leaves were compared with those in black teas (Figure 5). Key floral aroma compounds in withered leaves, such as linalool, α-cedrene, and nonanal, increased significantly after S, YLS, and HYS treatments (Figure 5A–C), while trans-β-ionone was not detected in withered leaves (Figure 5D). The content of these floral aroma compounds in both withered leaves and dry black teas was highest in YLS, suggesting that yellow-light withering further enhances the accumulation of floral substances on the basis of shaking, thereby improving black tea aroma quality. Benzeneacetaldehyde, 3-methyl-butanal, dimethyl sulfide, and cedrol also increased significantly under different shaking combinations in both withered leaves and dry black teas (Figure 5E–H), with YLS promoting the highest accumulation of benzeneacetaldehyde 3-methyl-butanal and cedrol and S promoting the highest accumulation of dimethyl sulfide and cedrol. The accumulation of these key aroma components in the withering process lays the foundation for the formation of tea aroma in the subsequent processing, thus facilitating the improvement in tea aroma quality.

## 4. Discussion

Aroma is a pivotal factor in tea evaluation, directly influencing tea pricing and consumer preference. Enhancing aroma quality through innovative processing techniques is one of the most effective strategies in tea production. Withering, the first and indispensable step in black tea processing, plays a crucial role in flavor formation, as volatile components are significantly generated during this stage [11,20]. During withering, photosynthesis, respiration, and most metabolic activities in tea leaves continue [9,17], and these processes can be modulated by postharvest treatments such as shaking and light irradiation. Previous research has demonstrated that introducing shaking procedures can significantly improve black tea quality, particularly its aroma [14,15,25]. Consistent with these findings, this study revealed that shaking and its combinations increased the content of alcohols, aldehydes, olefins, esters, and other compounds in black tea, promoting the enrichment of floral aroma volatiles and enhancing overall aroma quality. However, the effects varied across different shaking combinations.

Among the treatments, yellow-light withering combined with shaking (YLS) exhibited the most significant improvement in volatile compound formation, with the highest content of floral aroma substances in both withered leaves and dry black teas. This finding underscores the critical role of light quality in enhancing tea aroma and volatile metabolites [18,26,27,28]. It also suggests that yellow-light withering can further amplify the floral aroma quality of shaken black tea, aligning with previous studies that supplementary LED light during the standing stage of shaking promotes volatile formation in oolong tea [29]. A synergistic effect between yellow-light withering and shaking is hypothesized. During the yellow-light withering of the YLS treatment, yellow light may upregulate genes associated with volatile synthesis, such as linalool synthase genes (CsLIS2, CsLIS3, and CsLIS4) and farnesene synthase genes (CsFS5 and CsFS10), which have been reported to significantly increase under yellow light [30] and, thus, promote an increase in the content of related volatiles. As a cold light source, yellow light minimally affects leaf temperature, allowing tea leaves to remain fresh and responsive to external stresses like dehydration and mechanical damage during subsequent shaking. The dehydration and mechanical stress from shaking activate genes in volatile-related metabolic pathways [31], alters protein expression, and enhances enzyme activities that modulate volatile formation [32,33], thereby enriching the aroma components of black tea. Thus, yellow light may play a role in the early withering stage, while shaking contributes to the later stage, collectively promoting the accumulation of aroma substances to a greater extent.

In contrast, high-temperature withering combined with shaking (HTS) seemed to show an antagonistic effect. Although the aroma scores and total aroma content of HTS were higher than those of the control (CK), the content of many volatile constituents, including key aroma components such as linalool and α-cedrene, decreased after withering. Moreover, the total aroma content of HTS was lower than that of S (shaking alone), suggesting that high-temperature withering may counteract the positive effects of shaking. 

High-temperature withering accelerates water loss in tea leaves, potentially disrupting the activities of oxidase, hydrolase, and other enzymes involved in aroma anabolism [34]. For instance, higher air temperatures have been shown to decrease polyphenol oxidase activity while enhancing peroxidase activity in tea leaves [35]. Therefore, changes in enzyme activity may be the key to antagonism between shaking and high-temperature withering. Interestingly, the volatile content in HYS (high-temperature with yellow-light withering plus shaking) was similar to that of S, likely due to the counteracting effects of yellow light (which increases volatiles) and high temperature (which decreases volatiles). However, the precise mechanisms by which multiple treatments synergistically enhance black tea aroma quality require further investigation.

The concentration of volatile compounds alone is not sufficient to determine their significant contribution to the overall aroma. However, our study did not conduct specialized olfactory measurements for individual compounds. In order to evaluate the contribution of specific volatiles to the overall aroma of black tea, relative odor activity values (rOAVs) were calculated based on the odor threshold and the descriptions of specific compound odor characteristics are based on references from previous literature [28]. In this study, eight volatiles were identified as the most important aroma components (rOAVs > 1). To better understand the mechanisms underlying volatile changes during different withering treatments, these eight key compounds were classified into four categories based on their sources: volatile terpenes (VTs) (e.g., linalool, cedrol, and α-cedrene), amino acid-derived volatiles (AADVs) (e.g., benzeneacetaldehyde, 3-methyl-butanal, and dimethyl sulfide), carotenoid-derived volatiles (CDVs) (e.g., trans-β-ionone), and others (e.g., nonanal). Linalool can also be derived from the hydrolysis of glycosidically bound volatiles (GBVs) [36]. The YLS treatment showed the highest content of key VTs and AADVs, suggesting that YLS may enhance tea aroma by regulating terpenoid metabolism and amino acid degradation. VTs in tea leaves are synthesized via the methyl-erythritol phosphate (MEP) and mevalonic acid (MVA) pathways. Previous research has shown that light quality (e.g., red and yellow), and shaking can significantly upregulate genes associated with VT formation [18,26]. The higher content of VTs in YLS compared to S indicates that yellow light and shaking may synergistically regulate terpenoid metabolism, with yellow light promoting VT accumulation in the early withering stage and shaking enhancing it in the later stage. Similarly, amino acids undergo Strecker degradation under thermal conditions, producing key Strecker aldehydes such as 3-methyl-butanal, dimethyl sulfide, and benzeneacetaldehyde, with precursors that include leucine (Leu), methionine (Met), and phenylalanine (Phe), respectively [36]. Yellow-light withering [16,27] and shaking [25] have been shown to increase the content of free amino acids, which will provide abundant precursors for AADV formation and ultimately leading to higher concentrations in black tea.

The floral aroma is not only a hallmark of high-quality black tea but also a critical indicator of its excellence [37]. With the growing application of molecular sensory science in tea research, numerous studies have identified key aroma components responsible for the floral notes in black tea. For instance, indole and methyl jasmonate had been recognized as the most critical compounds determining the floral aroma intensity of Hunan black tea [15], while linalool, (E)-β-ionone, geraniol, β-myrcene, and (E)-2-hexenal, with high OAVs, have been identified as key floral volatiles in summer black tea [25]. Consistent with these findings, this study also identified linalool and trans-β-ionone as key floral components based on odor descriptions from **previous literature** [5]. Additionally, α-cedrene and nonanal, which contribute to floral aromas [23], were found to play significant roles in enhancing the aroma quality of black tea. The contents of linalool, α-cedrene, and nonanal were significantly upregulated under different shaking combinations, particularly in the YLS treatment, in both withered tea leaves and dry black teas. Trans-β-ionone, with its distinctive “violet-like” aroma [5], is a carotenoid-derived compound that can be produced either through enzymatic reactions during fermentation or thermal degradation during drying [36]. As a result, it was not detected in withered leaves, but its content in black tea increased remarkably in YLS-treated samples. Consistent with sensory evaluations, YLS, which exhibited the highest content of key floral components, also achieved the highest aroma score, characterized by a long-lasting floral aroma.

In conclusion, our findings highlight the significant impact of shaking and yellow-light withering on the aroma quality of black tea. The YLS treatment, in particular, demonstrated the most pronounced enhancement in floral aroma components, suggesting its potential as a valuable technique in black tea processing. Future research should integrate olfactory measurements and molecular sensory science to further elucidate the specific contributions of individual compounds to black tea aroma. This approach will provide a more comprehensive understanding of aroma formation and enable the development of targeted strategies for producing high-quality black tea.

## 5. Conclusions

This study evaluated the aroma attributes of black teas processed with different shaking combinations through sensory evaluation and GC-MS analysis. Compared to CK, all shaking combinations resulted in a distinct floral aroma, with YLS showing the highest floral aroma intensity, volatile compound content, and number. Eight key aroma compounds, including linalool, cedrol, 3-methyl-butanal, trans-β-ionone, dimethyl sulfide, benzeneacetaldehyde, α-cedrene, and nonanal, were identified as the most important contributors to black tea aroma, with their content significantly increased after shaking combinations in withered leaves (except trans-β-ionone). YLS exhibited the highest content of floral volatiles with high rOAVs, indicating a synergistic effect between yellow light and shaking. In summary, the findings of this study enhance our understanding of the aroma formation of black tea under different shaking combinations and provide a theoretical basis and practical guidance for producing high-aroma black tea through optimized shaking and withering techniques.

## Figures and Tables

**Table 1:** Sensory evaluation of black tea under different shaking combinations.

Sample	Appearance	Infusion Color	Aroma	Taste	Infused Leaf	Total Score
CK	90.00 ± 0.00 a	90.23 ± 0.21 a	85.00 ± 0.41 d	88.23 ± 0.21 c	90.00 ± 0.00 a	88.24 ± 0.13 d
S	90.00 ± 0.00 a	90.32 ± 0.13 a	88.23 ± 0.21 b	90.17 ± 0.12 b	90.00 ± 0.00 a	89.64 ± 0.06 b
YLS	90.00 ± 0.00 a	90.67 ± 0.24 a	90.57 ± 0.33 a	92.23 ± 0.21 a	90.00 ± 0.00 a	90.88 ± 0.09 a
HTS	90.00 ± 0.00 a	88.50 ± 0.41 b	87.33 ± 0.24 c	90.07 ± 0.09 b	90.00 ± 0.00 a	89.20 ± 0.11 c
HYS	90.00 ± 0.00 a	84.23 ± 0.21 c	88.07 ± 0.09 b	87.13 ± 0.09 d	90.00 ± 0.00 a	88.08 ± 0.07 d

The numerical values presented in this table represent the evaluation scores (mean ± SD, n = 5 panelists)., which are graded on a 100-point scale. The total scores were computed with the following weightings: appearance constitutes 20%, infusion color accounts for 10%, aroma makes up 30%, taste represents 30%, and infused leaves contribute 10% [21]. Treatments: CK represents indoor natural withering, S represents shaking, YLS represents yellow light plus shaking, HTS represents high temperature plus shaking, HYS represents high temperature with yellow light plus shaking. Different letters in the same column indicate a significant difference between different withering treatments (*p* < 0.05).

**Table 2:** Relative odor activity values (rOAVs) of 8 key aroma compounds (rOAV> 1) in black teas under different shaking combinations.

Name of Odorants	OT (μg∙L^−1^)	Odor Characteristic	Average rOAV				
			CK	S	YLS	HTS	HYS
Linalool	0.22	Floral, sweet, grape-like, woody	4.72 ± 0.75 c	30.34 ± 11.08 b	54.78 ± 0.82 a	22.14 ± 1.71 b	23.20 ± 7.45 b
Cedrol	1.00	Cedarwood aroma	6.08 ± 2.65 c	19.16 ± 7.02 a	21.29 ± 2.96 a	7.50 ± 0.33 c	14.33 ± 4.30 ab
3-methyl-Butanal	0.50	Apple-like and chocolate-like flavor	2.53 ± 0.16 d	18.14 ± 5.07 ab	25.63 ± 4.82 a	8.19 ± 0.75 c	16.87 ± 3.13 b
Trans-β-Ionone	0.007	Violet-like, floral, and raspberry-like	16.29 ± 6.28 c	8.51 ± 3.60 c	65.13 ± 7.99 a	37.66 ± 3.77 b	78.50 ± 32.76 a
Dimethyl sulfide	0.33	Corn, sulfurous flavor	0.88 ± 0.22 c	12.35 ± 4.39 a	7.64 ± 0.61 a	2.91 ± 0.36 b	4.16 ± 1.00 b
Benzeneacetaldehyde	1.20	Hawthorne, honey, sweet	0.20 ± 0.04 c	1.02 ± 0.36 b	1.77 ± 0.03 a	0.82 ± 0.10 b	1.79 ± 0.24 a
α-Cedrene	2.13	Floral	0.89 ± 0.19 c	4.66 ± 3.20 abc	5.67 ± 0.46 a	1.66 ± 0.13 bc	3.68 ± 1.57 ab
Nonanal	1.10	Rose aroma, aroma of citrus flower, creamy aroma	0.27 ± 0.05 c	1.14 ± 0.69 ab	1.90 ± 0.07 a	0.80 ± 0.03 b	1.21 ± 0.76 ab

rOAV: calculated by the relative concentration of odorant in tea with OTs in water (mean ± SD, n = 3). OTs: odor threshold in water based on the literature [5,23,24]. Treatments: CK represents indoor natural withering, S represents shaking, YLS represents yellow light plus shaking, HTS represents high temperature plus shaking, and HYS represents high temperature with yellow light plus shaking. Different lowercase letters in a row indicate a significant difference between different treatments (*p* < 0.05).

## Data Availability

The original contributions presented in the study are included in the article/supplementary material; further inquiries can be directed to the corresponding author.

## Supplementary Materials

The following supporting information can be downloaded at: https://www.mdpi.com/article/10.3390/foods14050758/s1. Table S1: Volatile components and their content in black tea under different shaking combinations; Table S2: Relative Odor activity values (rOAV) and Odor characteristics of Volatile compounds in black teas under different withering combinations; Table S3: Volatile components and their content in withered tea leaves under different shaking combinations; Figure S1: EI mass spectra of key aroma compounds and their corresponding mass spectra from database; Figure S2: Figure S2 Total ion chromatogram of black tea samples under different withering combinations; Figure S3: Figure S3 Total ion chromatogram of withered leaves under different withering combinations.
