# Supplementary material for: The Combination of Shaking and Yellow-Light Withering Promote the Volatile Aroma Components and the Aroma Quality of Black Tea
Source: Foods. 2025 Feb 23;14(5):758. doi: 10.3390/foods14050758 (PMC11899712; doi:10.3390/foods14050758)
Supplement: Supplementary file 1 [file foods-14-00758-s001.zip › foods-3489764-supplementary.pdf]

Supplementary data

Table S1 Volatile components and their content in black tea under different shaking combinations (mean ± SD, n= 3)

	Substance	Categories	RI		Retention times (min)	Relative content (μg/g)				
			Calculated	NIST		CK	S	YLS	HTS	HYS
1	2-ethyl-1-Hexanol	Alcohols	1031	1031	12.91	N.d	N.d	1.11±0.14	N.d	N.d
2	Benzyl alcohol	Alcohols	1040	1036	13.21	N.d	0.70±0.16a	0.78±0.03a	0.30±0.16a	0.74±0.44a
3	Cis-Linalool oxide	Alcohols	1070	1071	14.34	0.29±0.05d	2.23±0.19b	3.12±0.32a	1.28±0.16c	2.15±0.11b
4	Trans-Linalool oxide (furanoid)	Alcohols	1084	1086	14.92	0.65±0.09d	4.83±0.48b	6.42±0.92a	2.75±0.35c	4.70±0.38b
5	Linalool	Alcohols	1097	1099	15.43	1.04±0.20c	6.67±3.00b	12.05±0.22a	4.87±0.46b	5.11±2.01b
6	Fenchyl alcohol	Alcohols	1108	1113	15.83	0.10±0.02d	0.45±0.17bc	0.53±0.05ab	0.20±0.03cd	0.75±0.24a
7	Phenylethyl Alcohol	Alcohols	1121	1116	16.07	0.67±0.33c	1.46±0.13b	1.70±0.19ab	0.75±0.38c	2.32±0.56a
8	Endo-Borneol	Alcohols	1163	1167	17.72	0.16±0.04c	1.04±0.13a	1.08±0.23a	0.41±0.04b	1.19±0.09a
9	Trans-Linalool 3,7-oxide	Alcohols	1168	1173	17.91	0.10±0.06a	0.13±0.04a	0.48±0.32a	0.19±0.05a	0.39±0.26a
10	Epoxylinalol	Alcohols	1173	1178	18.12	0.14±0.03a	0.42±0.06a	0.57±0.29a	0.25±0.08	0.65±0.32a
11	α -Terpineol	Alcohols	1188	1189	18.68	0.64±0.22b	2.82±0.71a	3.07±0.40a	1.21±0.07b	3.46±0.56a
12	2,5-Cyclooctadien-1-ol	Alcohols	888	N.f	7.97	N.d	N.d	0.09±0.05	N.d	N.d
13	Geraniol	Alcohols	1261	1255	21.17	0.17±0.13c	1.16±0.08a	1.10±0.48ab	0.67±0.18b	1.33±0.09a
14	Cedrol	Alcohols	1596	1598	31.54	6.08±3.25b	19.17±8.60a	21.29±3.63a	7.5±0.40b	14.33±5.27ab
15	Nonanal	Aldehydes	1102	1104	15.61	0.27±0.06c	1.14±0.85ab	1.91±0.08a	0.80±0.03b	1.22±0.93ab
16	β -Cyclocitral	Aldehydes	1217	1220	19.74	0.20±0.04b	2.86±2.82a	1.48±0.03ab	0.49±0.03a	1.03±0.44ab
17	Hexanal	Aldehydes	807	800	5.08	0.05±0.02c	2.67±1.70ab	3.22±2.10a	0.28±0.02bc	0.65±0.03bc

18	(E)-2-Hexenal	Aldehydes	841	854	6.77	0.08±0.03d	2.44±0.38a	2.29±0.61ab	0.84±0.20c	1.71±0.10b
19	Heptanal	Aldehydes	900	901	8.32	0.03±0.01b	0.28±0.08a	0.27±0.02a	0.13±0.02b	0.30±0.08a
20	Benzaldehyde	Aldehydes	967	962	10.53	0.07±0.02c	0.93±0.03a	0.93±0.27a	0.43±0.11b	1.00±0.28a
21	3-methyl-Butanal	Aldehydes	674	652	2.59	1.28±0.30c	9.07±3.11ab	12.81±2.95a	4.10±0.46c	8.43±1.92b
22	Benzeneacetaldehyde	Aldehydes	1044	1045	13.52	0.83±0.32c	4.07±1.77b	7.06±0.16a	3.26±0.49b	7.18±1.17a
23	2,6,6-trimethyl-1-Cyclohexene-1-acetaldehyde	Aldehydes	1254	1254	20.92	N.d	0.47±0.23a	N.d	0.27±0.03a	0.40±0.20a
24	Methyl salicylate	Esters	1195	1192	18.95	0.24±0.06c	0.54±0.14bc	3.23±0.37a	0.93±0.11b	0.30±0.17c
25	Cis-3-Hexenyl isovalerate	Esters	1231	1238	20.13	N.d	0.60±0.50ab	0.96±0.24a	0.42±0.04ab	0.32±0.21b
26	Geranyl isovalerate	Esters	1236	1585	20.3	N.d	N.d	N.d	0.32±0.05	N.d
27	L- α -bornyl acetate	Esters	1281	1285	21.89	N.d	N.d	0.54±0.01a	N.d	0.52±0.29a
28	α -Terpinyl acetate	Esters	1347	1350	24.04	0.35±0.14c	1.39±0.76ab	1.97±0.12a	0.76±0.07bc	1.73±0.81ab
29	Methyl myrtenate	Esters	1293	1301	22.31	0.04±0.02	0.19±0.08	N.d	0.11±0.02	0.21±0.10
30	3-Hydroxy-2,4,4-trimethylpentyl 2-methylpropanoate	Esters	1370	1373	24.81	0.11±0.05c	0.57±0.28a	0.47±0.08ab	0.19±0.20bc	0.48±0.11a
31	5,8,11-Heptadecatriynoic acid, methyl ester	Esters	1541	N.f	30.05	N.d	0.16±0.09ab	0.20±0.01a	0.06±0.01b	N.d
32	Cedryl acetate	Esters	1759	1762	36.12	N.d	0.25±0.16a	0.31±0.04a	0.12±0.01a	0.23±0.11a
33	(3Z)-3-Hexenyl 2-methylbutanoate	Esters	1236	1234	20.29	N.d	0.59±0.44a	N.d	N.d	0.19±0.12b
34	(Z)-Hexanoic acid, 3-hexenyl ester	Esters	1381	1380	25.14	0.13±0.05b	N.d	0.54±0.19a	0.27±0.18ab	0.13±0.05ab
35	β -Guaiene	Olefins	1495	1490	28.74	N.d	N.d	0.43±0.05	N.d	N.d
36	Cuparene	Olefins	1499	1505	28.87	0.12±0.05c	0.65±0.04a	0.66±0.05a	0.22±0.03bc	0.46±0.29ab
37	δ -Elemene	Olefins	1507	1515	29.09	N.d	N.d	1.06±0.13	N.d	N.d

38	(+)-δ-Cadinene	Olefins	1518	1524	29.4	0.27±0.10b	0.89±0.64ab	1.30±0.14a	0.41±0.05b	0.88±0.48ab
39	Ylangene	Olefins	1378	1372	25.08	N.d	0.19±0.15a	0.29±0.03a	N.d	N.d
40	β -Elemene	Olefins	1386	1391	25.35	0.17±0.03b	1.19±0.93ab	1.22±0.09a	0.43±0.07ab	1.12±0.59ab
41	α -Cedrene	Olefins	1404	1422	25.95	1.90±0.49d	6.82±5.40ab	12.06±1.20a	3.53±0.33c	7.84±4.08ab
42	β -Cedrene	Olefins	1413	1421	26.19	0.92±0.30b	3.02±2.35ab	5.10±0.50a	1.54±0.15b	3.27±1.81ab
43	Cis-Thujopsene	Olefins	1424	1429	26.53	0.19±0.05b	0.71±0.58ab	1.22±0.13a	0.36±0.06b	0.80±0.44ab
44	β -Copaene	Olefins	1459	1432	27.59	N.d	0.38±0.22a	0.44±0.05a	0.26±0.03a	0.36±0.20a
45	α -Pinene	Olefins	928	937	9.19	N.d	0.19±0.18a	0.30±0.08a	0.11±0.05a	N.d
46	D-Limonene	Olefins	1025	1018	12.69	N.d	0.49±0.03ab	0.57±0.09a	0.24±0.02b	0.49±0.25ab
47	Dimethyl sulfide	Organosulfur compounds	510	520	1.69	0.29±0.09c	4.07±1.77a	2.52±0.25ab	0.96±0.14bc	1.37±0.40bc
48	Trans-2-Hexenoic acid	Acids	767	805	3.70	N.d	N.d	N.d	0.10±0.06	N.d
49	Methyl N-hydroxybenzimidate (Oxime-, methoxy-phenyl-_)	Aromatic hydrocarbon	935	N.f	9.28	0.80±0.37b	3.36±2.58ab	4.32±2.13a	1.42±0.68ab	2.93±0.48ab
50	2-pentyl-Furan	Heterocyclic compounds	989	993	11.43	N.d	0.84±0.61ab	1.43±0.29a	0.49±0.10b	0.67±0.33ab
51	Geranyl vinyl ether	Ethers	1274	1250	21.62	N.d	N.d	0.04±0.01	N.d	N.d
52	trans- β -Ionone	Ketones	1483	1486	28.37	0.11±0.06c	0.06±0.03c	0.45±0.07ab	0.26±0.03bc	0.55±0.28a
53	β -Ionone	Ketones	1480	1491	28.28	0.13±0.04b	0.05±0.04b	0.55±0.08a	0.23±0.03ab	0.50±0.38a

RI represents retention index. N.f denotes that the RI cannot be found from the National Institute of Standards and Technology mass spectral database (NIST 17). N.d denotes that the component was not detected in the sample. Different lowercase letters above the bars in the same volatile category indicate significant differences at $p < 0.05$. Treatments: CK represents indoor natural withering, S represents shaking, YLS represents yellow light plus shaking, HTS represents high temperature plus shaking, HYS represents high temperature with yellow light plus shaking.

Table S2 Relative odor activity values (rOAV) and Odor characteristics of Volatile compounds in different black teas (mean ± SD, n= 3)

	Name of odorants	OT (μg·kg^{-1} in water)	Odor characteristic	Average rOAV				
				CK	S	YLS	HTS	HYS
1.	Linalool	1.00	Floral, sweet, grape-like, woody	4.72±0.75	30.34±11.08	54.78±0.82	22.14±1.71	23.20±7.45
2.	Cedrol	0.22	Cedarwood aroma	6.08±2.65	19.16±7.02	21.29±2.96	7.50±0.33	14.33±4.30
3.	3-methyl-Butanal	0.50	Apple-like and chocolate-like flavor under high dilution	2.53±0.16	18.14±5.07	25.63±4.82	8.19±0.75	16.87±3.13
4.	Trans-β-Ionone	0.007	Violet-like, floral, and raspberry-like	16.29±6.28	8.51±3.60	65.13±7.99	37.66±3.77	78.50±32.76
5.	Dimethyl sulfide	0.33	Corn, sulphurous flavour	0.88±0.22	12.35±4.39	7.64±0.61	2.91±0.36	4.16±1.00
6.	Benzeneacetaldehyde	1.20	Hawthorne, honey, sweet	0.20±0.04	1.02±0.36	1.77±0.03	0.82±0.10	1.79±0.24
7.	α -Cedrene	2.13	floral	0.89±0.19	4.66±0.20	5.67±0.46	1.66±0.13	3.68±1.57
8.	Nonanal	1.00	Rose aroma, aroma of citrus flower, creamy aroma	0.27±0.05	1.14±0.69	1.90±0.07	0.80±0.03	1.21±0.76
9.	β-Cyclocitral	3.00	Herbal, clean, rose-like, fruity	0.07±0.01	0.95±0.77	0.49±0.01	0.16±0.01	0.34±0.12
10.	Hexanal	4.5	Grassy, green, fresh, fatty	0.01±0.00	0.59±0.31	0.72±0.38	0.06±0.00	0.14±0.01
11.	Cis-Linalool oxide	6.00	Fruity aroma	0.05±0.01	0.37±0.03	0.52±0.04	0.21±0.02	0.36±0.01
12.	2-pentyl-Furan	6.00	Butter, floral, fruit	N.d	0.14±0.08	0.24±0.04	0.08±0.00	0.11±0.04
13.	Geraniol	7.50	Rose-like, sweet, honey-like	0.02±0.01	0.15±0.01	0.15±0.05	0.09±0.02	0.18±0.01
14.	β -Ionone	7.00	Floral aroma, fruity fragrance	0.00±0.00	0.30±0.09	0.24±0.04	0.31±0.05	0.41±0.06
15.	Heptanal	5.40	Penetrating fruity odor	0.02±0.00	0.01±0.00	0.08±0.01	0.03±0.00	0.07±0.04
16.	Trans-Linalool oxide (furanoid)	190.00	Sweet, floral, creamy	0.00±0.00	0.03±0.00	0.03±0.00	0.01±0.00	0.02±0.00
17.	D-Limonene	10.00	Eucalyptus, lemongrass, citrus	N.d	0.05±0.00	0.06±0.01	0.02±0.00	0.05±0.02
18.	(E)-2-Hexenal	110.00	Green, leafy, fruity	0.00±0.00	0.02±0.00	0.02±0.00	0.01±0.00	0.02±0.00
19.	Methyl salicylate	40.00	Wintergreen aroma	0.01±0.00	0.01±0.00	0.08±0.01	0.02±0.00	0.01±0.00
20.	(Z)-Hexanoic acid, 3-hexenyl ester	16.00	Green, waxy, winey, grassy	0.01±0.00	N.d	0.03±0.01	0.02±0.01	0.02±0.01
21.	α -Terpineol	300	Anise, Fresh, Mint, Oil	0.00±0.00	0.01±0.00	0.01±0.00	0.00±0.00	0.01±0.00
22.	Benzyl alcohol	100.00	Faint aromatic, fruity odour	N.d	0.00±0.00	0.00±0.00	0.00±0.00	0.00±0.00
23.	Phenylethyl Alcohol	390	Fruit, Honey, Lilac, Rose, Wine	0.00±0.00	0.00±0.00	0.00±0.00	0.00±0.00	0.01±0.00
24.	Benzaldehyde	350	Bitter almond-like	N.d	0.00±0.00	0.00±0.00	0.00±0.00	0.00±0.00

rOAV: calculated by the relative concentration of odorant in tea with OTs in water. OTs: odor threshold in water based on the literature (Hou et al. 2020, Xie et al. 2021, Guo et al. 2022, Xie et al. **2023). Treatments:** CK represents indoor natural withering, S represents shaking, YLS represents yellow light plus shaking, HTS represents high temperature plus shaking, HYS represents high temperature with yellow light plus shaking. Different lowercase letters in a row indicate a significant difference between different treatments ($p < 0.05$).

Table S3: Volatile components and their content in withered tea leaves under different shaking combinations (mean ± SD, n= 3)

No.	Substance	RI		Retention time（min）	Relative contens（μg/g）				
		Calculated	NIST		CK	S	YLS	HTS	HYS
	Cyclopentanol	747	768	3.15	0.02±0.00	N.d	N.d	N.d	N.d
	(Z)-2-Penten-1-ol	780	767	4.87	0.01±0.00a	0.01±0.00a	0.01±0.00a	0.01±0.00a	0.01±0.00a
	(E)-3-Hexen-1-ol	860	856	6.94	0.29±0.04ab	0.36±0.07a	0.31±0.06ab	0.09±0.01c	0.25±0.04b
	3-methyl-4-Penten-1-ol	865	N.f	7.37	0.11±0.01c	0.18±0.02a	0.19±0.03a	0.04±0.01d	0.15±0.01b
	Benzyl alcohol	1045	1036	13.58	0.11±0.01b	0.15±0.01a	0.11±0.00b	0.03±0.01c	0.10±0.01b
	Cis-Linalool oxide	1073	1074	14.49	1.18±0.03b	1.25±0.02ab	1.18±0.05b	0.38±0.01c	1.31±0.09a
	trans-Linalool oxide (furanoid)	1090	1086	15.14	2.65±0.11c	2.94±0.06ab	2.83±0.10b	0.85±0.04d	3.06±0.06a
	Linalool	1105	1099	15.74	4.55±0.11c	4.97±0.19b	5.83±0.34a	1.42±0.03d	5.22±0.13b
	Fenchyl alcohol	1115	1113	16.04	N.d	0.02±0.00	N.d	N.d	N.d
[illegible]	Phenylethyl Alcohol	1118	1116	16.17	0.43±0.0c1	0.82±0.07ab	0.75±0.04b	0.22±0.02d	0.90±0.07a
[illegible]	endo-Borneol	1167	1167	17.87	N.d	N.d	N.d	0.02±0.01a	0.11±0.05a
[illegible]	trans-Linalool 3,7-oxide	1171	1173	18.05	0.27±0.02b	0.47±0.01a	0.45±0.01a	0.12±0.01c	0.47±0.03a
[illegible]	Epoxylinalol	1177	1178	18.28	0.30±0.01d	0.58±0.02a	0.51±0.02c	0.14±0.01e	0.58±0.04b
[illegible]	2-Methylenecholestan-3-ol	1238	1238	20.37	N.d	0.01±0.00	N.d	N.d	N.d
[illegible]	α- Terpineol	1192	1189	18.83	0.26±0.01c	0.44±0.02a	0.44±0.02a	0.10±0.01d	0.37±0.02b
[illegible]	Geraniol	1259	1255	21.09	0.17±0.01c	0.29±0.03ab	0.25±0.04b	0.10±0.02d	0.34±0.04a
[illegible]	(±)-trans-Nerolidol	1565	1564	30.75	0.04±0.01b	0.08±0.01a	0.06±0.01a	0.01±0.01c	0.07±0.01a
[illegible]	Cedrol	1602	1598	31.86	1.23±0.09b	1.59±0.05a	1.48±0.08a	0.29±0.02d	0.90±0.05c
[illegible]	3-methyl-Butanal	675	652	2.66	N.d	0.05±0.01a	0.05±0.01a	N.d	0.05±0.02a
[illegible]	Benzeneacetaldehyde	1048	1045	13.52	N.d	N.d	0.18±0.01a	0.05±0.01b	0.17±0.00a
[illegible]	Nonanal	1106	1104	15.78	N.d	N.d	0.03±0.01	N.d	0.02±0.00
[illegible]	Butanal	614	593	1.95	N.d	N.d	0.03±0.01	N.d	N.d
[illegible]	Benzaldehyde	972	962	10.78	N.d	0.02±0.00a	0.02±0.01a	N.d	N.d
[illegible]	Hexanal	807	800	5.32	N.d	N.d	0.02±0.00a	N.d	0.01±0.01a
[illegible]	(E)-2-Hexenal	842	854	6.8	N.d	0.01±0.00b	0.02±0.01a	N.d	0.02±0.01ab
[illegible]	Lilac aldehyde D	1153	1169	17.38	N.d	0.02±0.01a	0.02±0.00a	N.d	N.d
[illegible]	β-Cyclocitral	1220	1220	19.79	N.d	0.02±0.00a	0.02±0.00a	0.01±0.0b	0.02±0.00a
[illegible]	D-Limonene	1029	1030	12.82	0.01±0.01b	0.01±0.00b	N.d	0.01±0.00b	0.02±0.00a
[illegible]	α-Cedrene	1410	1422	26.10	0.63±0.03b	0.75±0.00a	0.59±0.02c	0.13±0.01d	0.50±0.01c
[illegible]	β-Cedrene	1418	1414	26.34	0.30±0.01b	0.36±0.01a	0.28±0.01b	0.05±0.03d	0.23±0.01c
[illegible]	Cis-Thujopsene	1429	1429	26.67	0.06±0.01b	0.08±0.00a	0.06±0.00b	0.01±0.00d	0.05±0.00c
[illegible]	(Z)-beta-Elemene	1390	1391	25.47	0.05±0.00	N.d	N.d	N.d	N.d
[illegible]	α-Curcumene	1482	1483	28.33	N.d	0.03±0.00	N.d	N.d	N.d
[illegible]	Cuparene	1503	1505	28.99	N.d	0.03±0.01a	0.03±0.00ab	0.01±0.01c	0.02±0.01b
[illegible]	(3aS,8aS)-6,8a-Dimethyl-3-(propan-2-ylidene)-1,2,3,3a,4,5,8,8a-octahydroazulene	1511	1532	29.21	0.06±0.00a	0.08±0.00a	N.d	N.d	N.d

(+)-δ-Cadinene	1522	1524	29.53	0.09±0.00b	0.11±0.01a	0.08±0.01b	0.02±0.00d	0.07±0.01c
(E)-3-Hexen-1-ol, acetate,	1011	1005	12.22	N.d	0.01±0.01a	N.d	0.01±0.00a	±
Hexanoic acid, hexyl ester	1386	1391	25.34	0.12±0.01a	0.13±0.01a	0.10±0.01b	0.02±0.01c	0.10±0.00b
(E)-Hexanoic acid, 2-hexenyl ester	1390	1391	25.46	N.d	0.24±0.01a	0.20±0.01b	0.04±0.01d	0.16±0.01c
(E)-Butanoic acid, 3-hexenyl ester	1188	1185	18.67	0.12±0.01d	0.26±0.01b	0.20±0.01c	0.05±0.01e	0.29±0.01a
Methyl salicylate	1195	1192	18.95	0.56±0.04ab	0.57±0.03a	0.51±0.02b	0.16±0.01c	0.55±0.02ab
Cis-3-Hexenyl-α-methylbutyrate	1234	1234	20.23	0.27±0.01d	0.54±0.02a	0.36±0.01c	0.09±0.01e	0.39±0.00b
Cis-3-Hexenyl isovalerate	1240	1238	20.44	0.09±0.00c	0.10±0.0b1	N.d	0.03±0.01d	0.12±0.00a
2-methyl-Butanoic acid, hexyl ester	1238	1236	20.37	N.d	0.13±0.01a	0.09±0.01b	N.d	N.d
Trans-2-Hexenyl isovalerate	1240	1245	20.44	N.d	N.d	0.08±0.00a	0.02±0.00a	0.06±0.00a
Acetic acid, 1,7,7-trimethyl-bicyclo [2.2.1]hept-2-yl ester	1285	1286	22.01	0.02±0.00a	0.03±0.00a	0.03±0.00a	0.01±0.00a	0.03±0.00a
α-Terpinyl acetate	1350	1350	24.14	0.12±0.01b	0.17±0.01a	0.16±0.01a	0.03±0.01c	0.12±0.01b
7-Methyl-Z-tetradecen-1-ol acetate	1363	N.f	24.58	N.d	0.01±0.00	N.d	N.d	N.d
Propanoic acid, 2-methyl-, 3-hydroxy-2,2,4-trimethylpentyl ester	1373	1380	24.91	0.04±0.01c	0.06±0.01a	0.07±0.00a	0.02±0.00d	0.05±0.01b
(Z)-Hexanoic acid, 3-hexenyl ester	1233	1234	25.18	0.25±0.00d	0.49±0.02a	0.40±0.01c	0.09±0.01e	0.43±0.01b
Geranyl vinyl ether	1225	N.f	19.98	0.01±0.00a	0.01±0.00a	0.01±0.00a	N.d	0.01±0.00a
Dimethyl sulfide	510	520	1.84	0.04±0.02b	0.12±0.06a	N.d	N.d	N.d

RI represents retention index. N.f denotes that RI cannot be found from the National Institute of Standards and Technology mass spectral database (NIST 17). N.d denotes that the component was not detected in the sample. Different lowercase letters above the bars in the same volatile category indicate significant differences at $p < 0.05$. Treatments: CK represents indoor natural withering, S represents shaking, YLS represents yellow light plus shaking, HTS represents high temperature plus shaking, HYS represents high temperature with yellow light plus shaking.

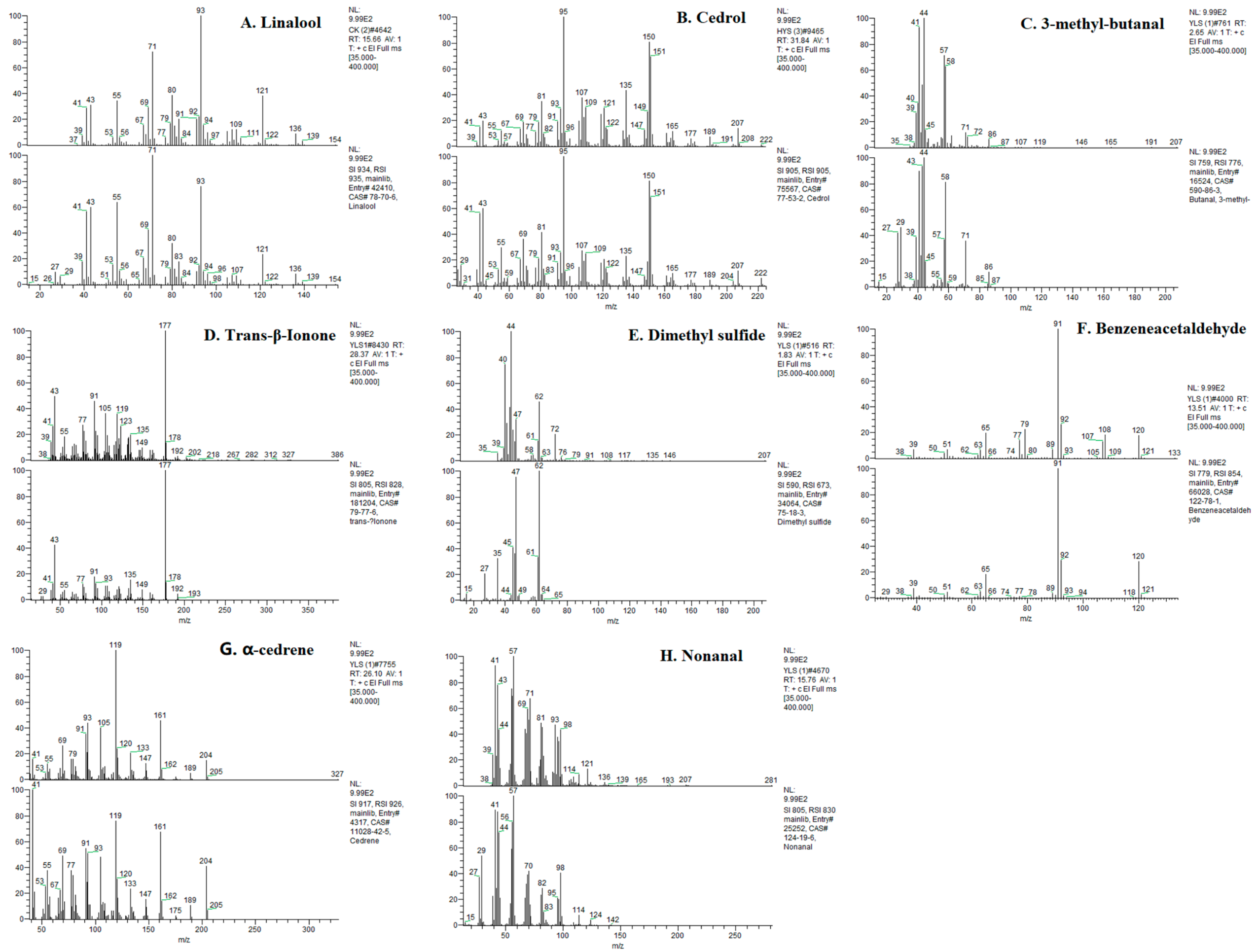

Figure S1 EI mass spectra of key aroma compounds and their corresponding EI mass spectra from database: (A) Linalool, (B) Cedrol, (C) 3-methyl-Butanal, (D) Trans-β-Ionone, (E) Dimethyl sulfide, (F) Benzeneacetaldehyde, (G) α-Cedrene, and (H) Nonanal.

Figure S2 Total ion chromatogram of black tea samples under different withering combinations

Chromatograms were as followed in an order of CK, S, YLS, HTS and HYS:

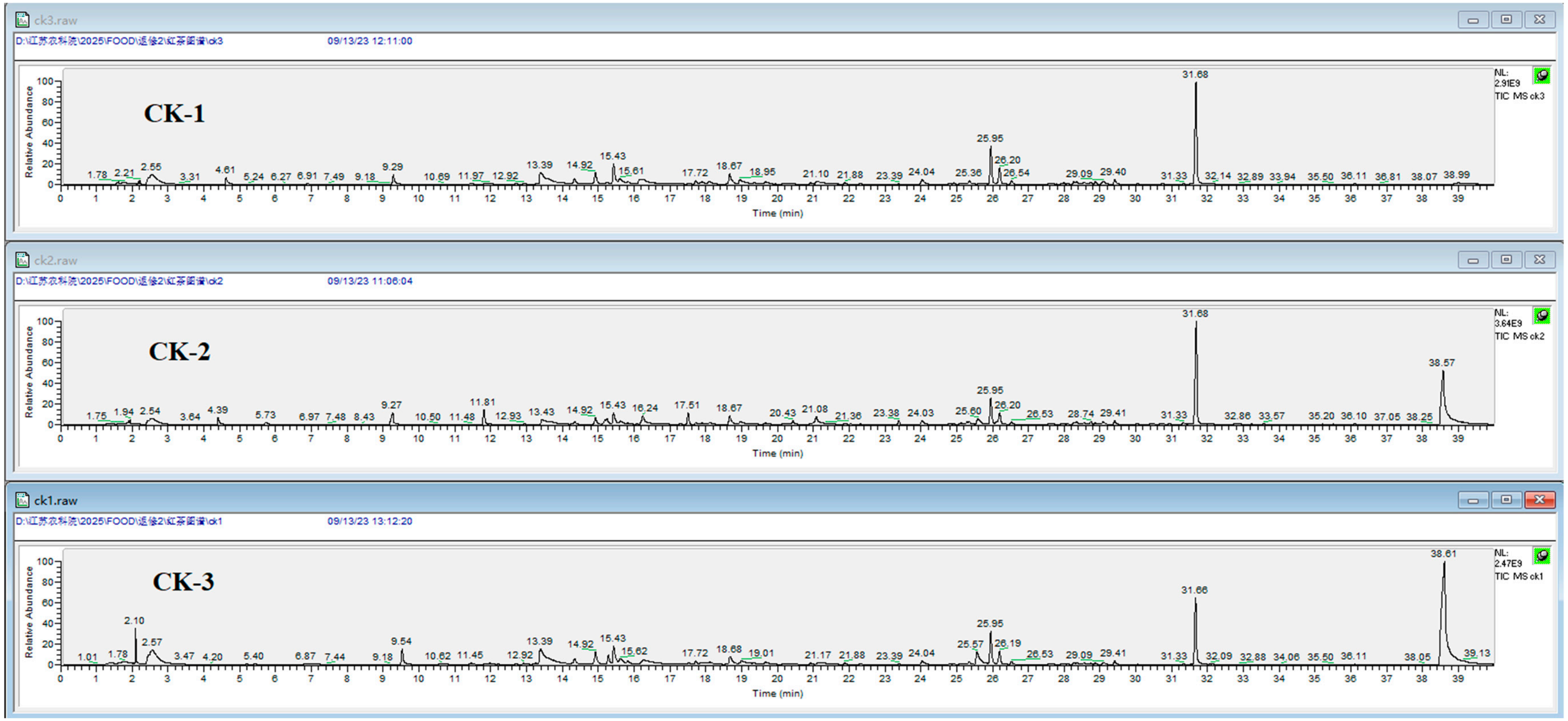

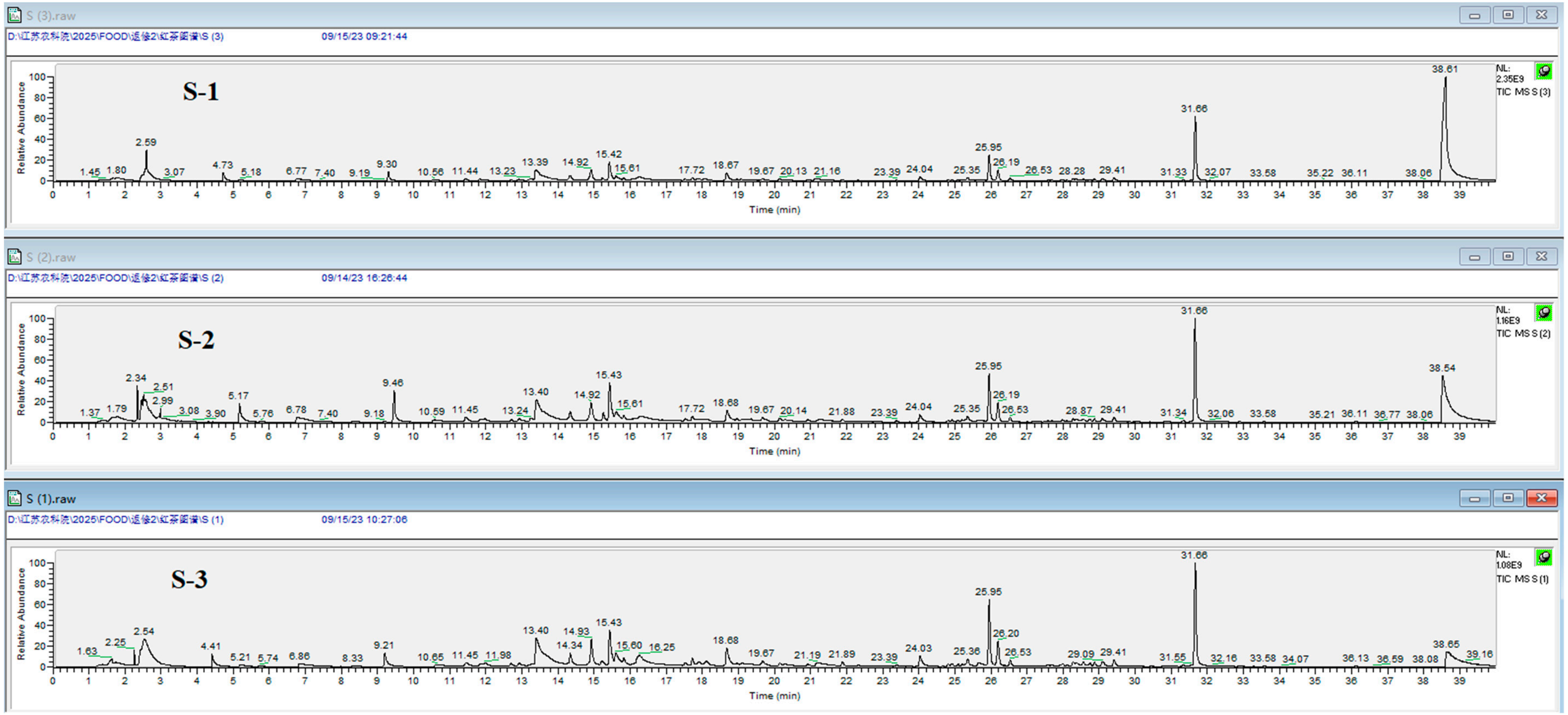
S (3).raw
D:\江苏农科院\2025\FOOD\返修2\红茶图谱\S (3)
09/15/23 09:21:44
S-1
NL: 2.35E9
TIC MS S (3)
1.45 1.80 2.59 3.07 4.73 5.18 6.77 7.40 9.19 9.30 10.56 11.44 13.23 13.39 14.92 15.42 15.61 17.72 18.67 19.67 20.13 21.16 23.39 24.04 25.35 25.95 26.19 26.53 28.28 29.41 31.33 31.66 32.07 33.58 35.22 36.11 38.06 38.61
Relative Abundance
Time (min)
S (2).raw
D:\江苏农科院\2025\FOOD\返修2\红茶图谱\S (2)
09/14/23 16:26:44
S-2
NL: 1.16E9
TIC MS S (2)
1.37 1.79 2.34 2.51 2.99 3.08 3.90 5.17 5.76 6.78 7.40 9.18 9.46 10.59 11.45 13.24 13.40 14.92 15.43 15.61 17.72 18.68 19.67 20.14 21.88 23.39 24.04 25.35 25.95 26.19 26.53 28.87 29.41 31.34 31.66 32.06 33.58 35.21 36.11 36.77 38.06 38.54
Relative Abundance
Time (min)
S (1).raw
D:\江苏农科院\2025\FOOD\返修2\红茶图谱\S (1)
09/15/23 10:27:06
S-3
NL: 1.08E9
TIC MS S (1)
1.63 2.25 2.54 4.41 5.21 5.74 6.86 8.33 9.21 10.65 11.45 11.98 13.40 14.34 14.93 15.43 15.60 16.25 18.68 19.67 21.19 21.89 23.39 24.03 25.36 25.95 26.20 26.53 29.09 29.41 31.55 31.66 32.16 33.58 34.07 36.13 36.59 38.08 38.65 39.16
Relative Abundance
Time (min)

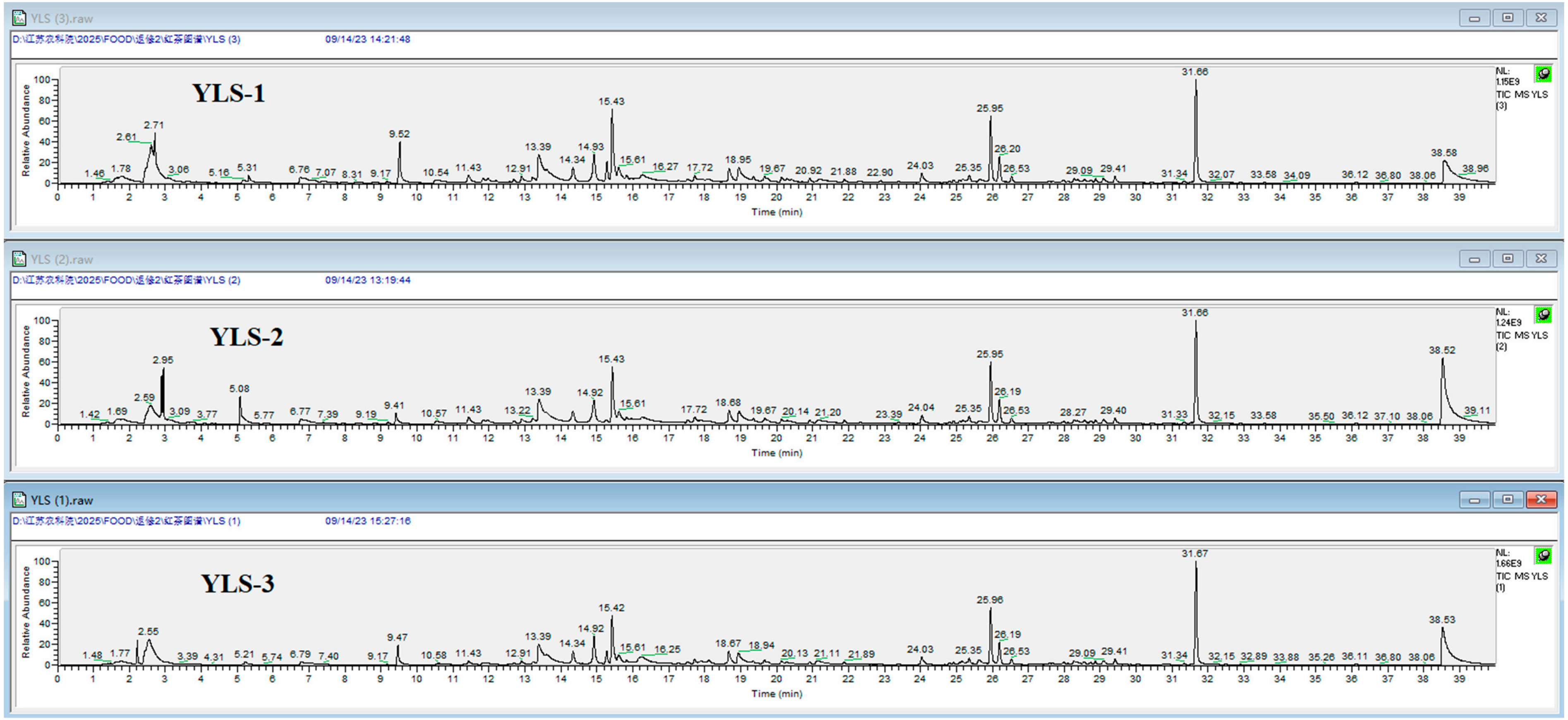
YLS (3).raw
09/14/23 14:21:48
YLS-1
NL: 1.15E9 TIC MS YLS (3)
YLS (2).raw
09/14/23 13:19:44
YLS-2
NL: 1.24E9 TIC MS YLS (2)
YLS (1).raw
09/14/23 15:27:16
YLS-3
NL: 1.66E9 TIC MS YLS (1)
Relative Abundance
Time (min)

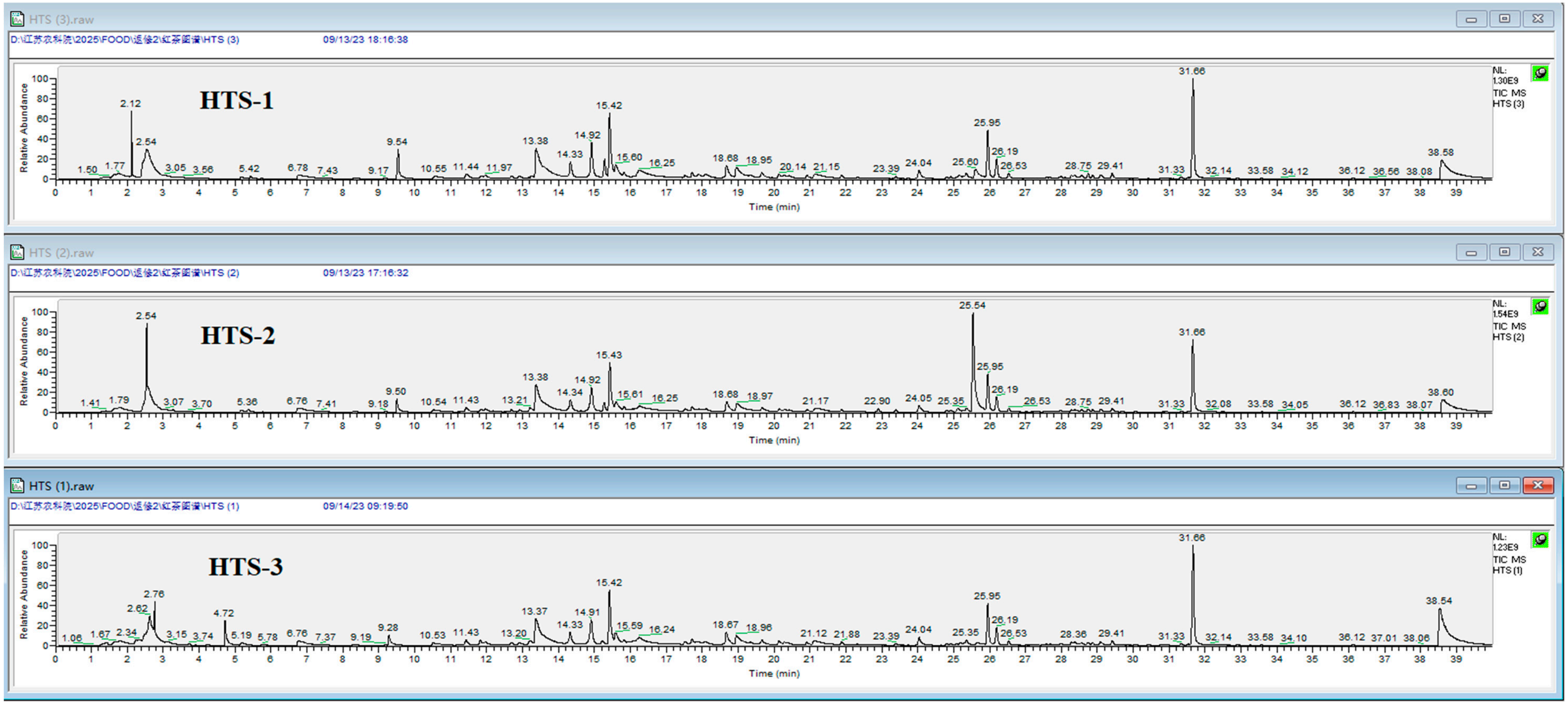
HTS (3).raw
D:\江苏农科院\2025\FOOD\返修2\红茶图谱\HTS (3)
09/13/23 18:16:38
HTS-1
NL: 1.30E9 TIC MS HTS (3)
2.12 2.54 9.54 13.38 14.33 14.92 15.42 15.60 16.25 18.68 18.95 24.04 25.60 25.95 26.19 26.53 28.75 29.41 31.33 31.66 32.14 33.58 34.12 36.12 36.56 38.08 38.58
Relative Abundance
Time (min)
HTS (2).raw
D:\江苏农科院\2025\FOOD\返修2\红茶图谱\HTS (2)
09/13/23 17:16:32
HTS-2
NL: 1.54E9 TIC MS HTS (2)
2.54 9.50 13.38 14.34 14.92 15.43 15.61 16.25 18.68 18.97 24.05 25.35 25.54 25.95 26.19 26.53 28.75 29.41 31.33 31.66 32.08 33.58 34.05 36.12 36.83 38.07 38.60
Relative Abundance
Time (min)
HTS (1).raw
D:\江苏农科院\2025\FOOD\返修2\红茶图谱\HTS (1)
09/14/23 09:19:50
HTS-3
NL: 1.23E9 TIC MS HTS (1)
2.62 2.76 4.72 9.28 13.37 14.33 14.91 15.42 15.59 16.24 18.67 18.96 24.04 25.35 25.95 26.19 26.53 28.36 29.41 31.33 31.66 32.14 33.58 34.10 36.12 37.01 38.06 38.54
Relative Abundance
Time (min)

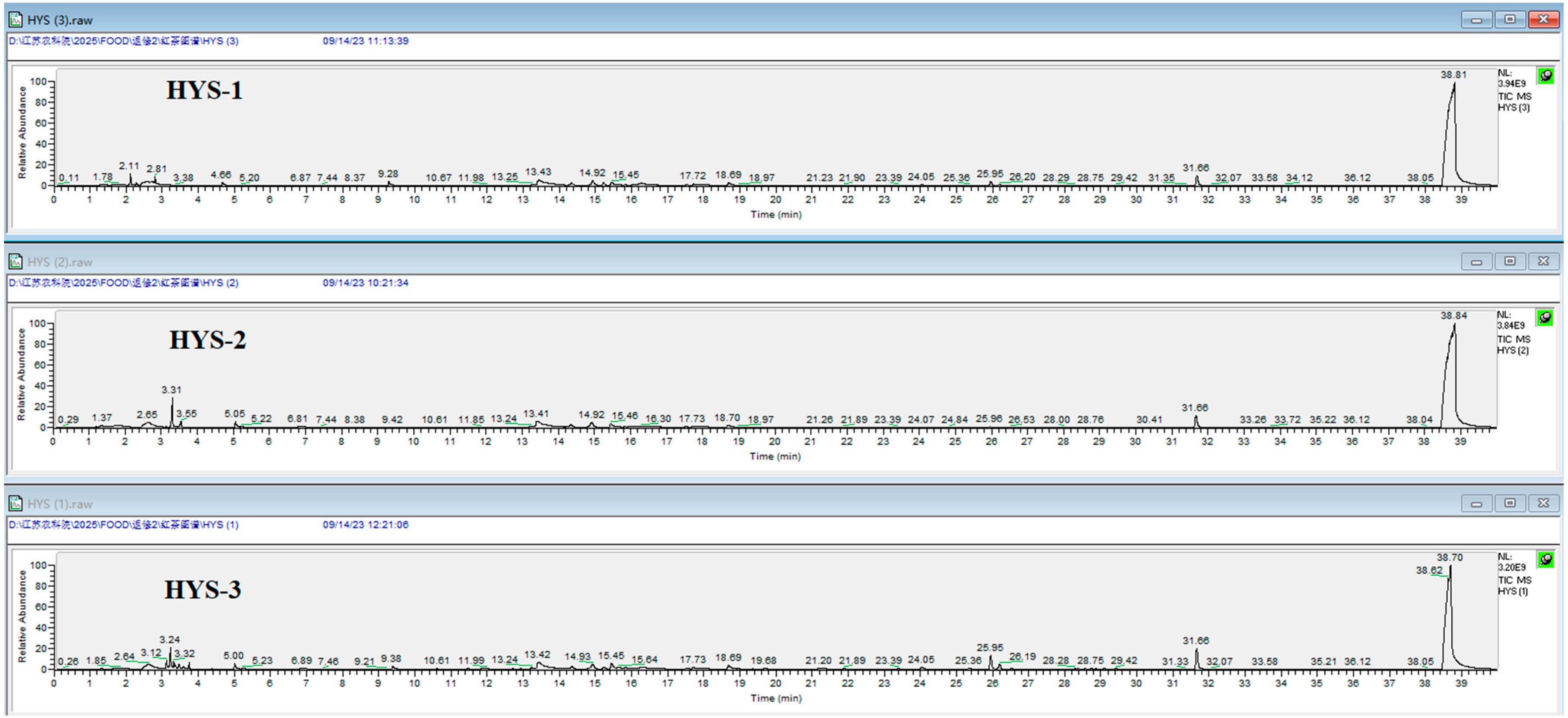
HYS (3).raw
D:\江苏农科院\2025\FOOD\返修2\红茶图谱\HYS (3)
09/14/23 11:13:39
HYS-1
NL: 3.94E9 TIC MS HYS (3)
38.81
31.66
Relative Abundance
Time (min)
HYS (2).raw
D:\江苏农科院\2025\FOOD\返修2\红茶图谱\HYS (2)
09/14/23 10:21:34
HYS-2
NL: 3.84E9 TIC MS HYS (2)
38.84
3.31
31.66
Relative Abundance
Time (min)
HYS (1).raw
D:\江苏农科院\2025\FOOD\返修2\红茶图谱\HYS (1)
09/14/23 12:21:06
HYS-3
NL: 3.20E9 TIC MS HYS (1)
38.70
38.62
3.24
25.95
31.66
Relative Abundance
Time (min)

Figure S3 Total ion chromatogram of withered leaves under different withering combinations

Chromatograms were as followed in an order of CK, S, YLS, HTS and HYS:

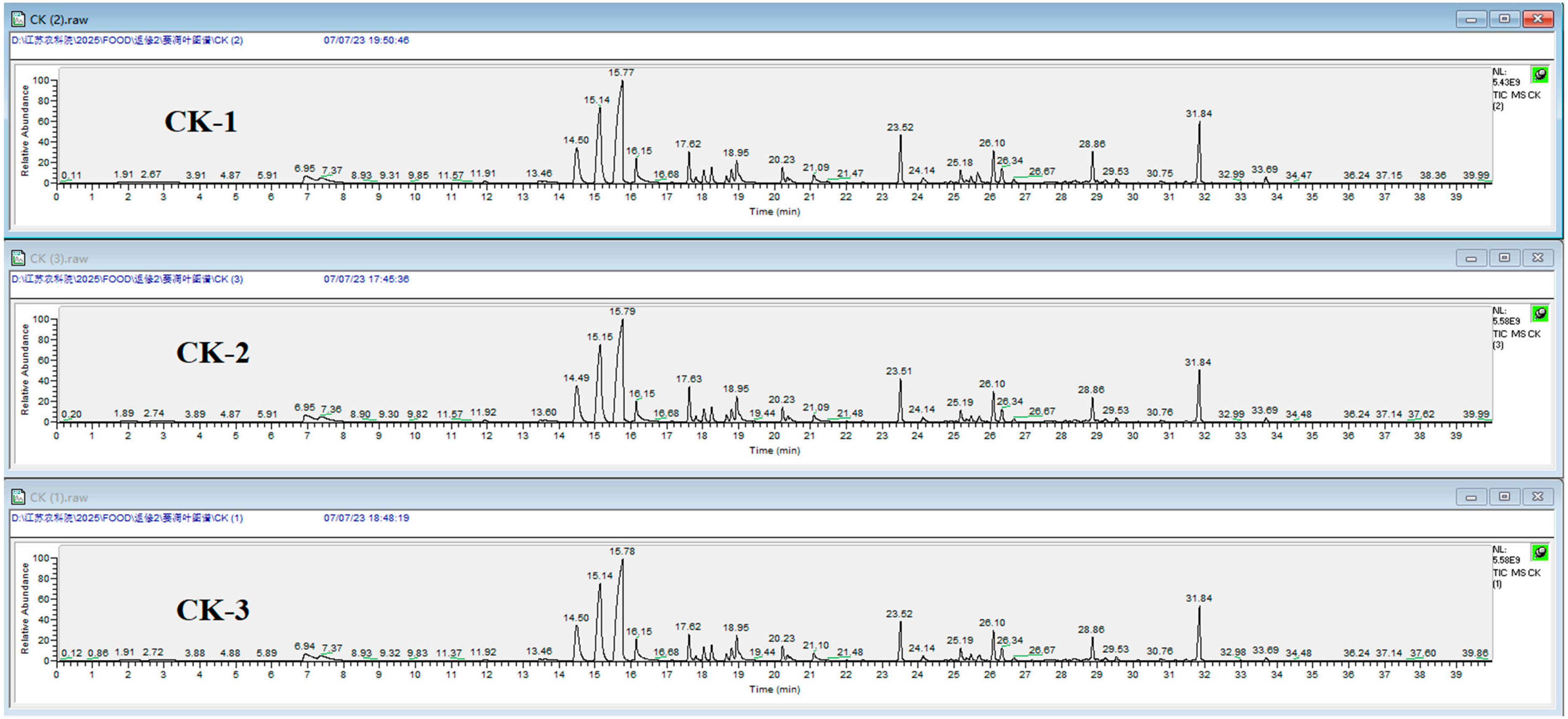

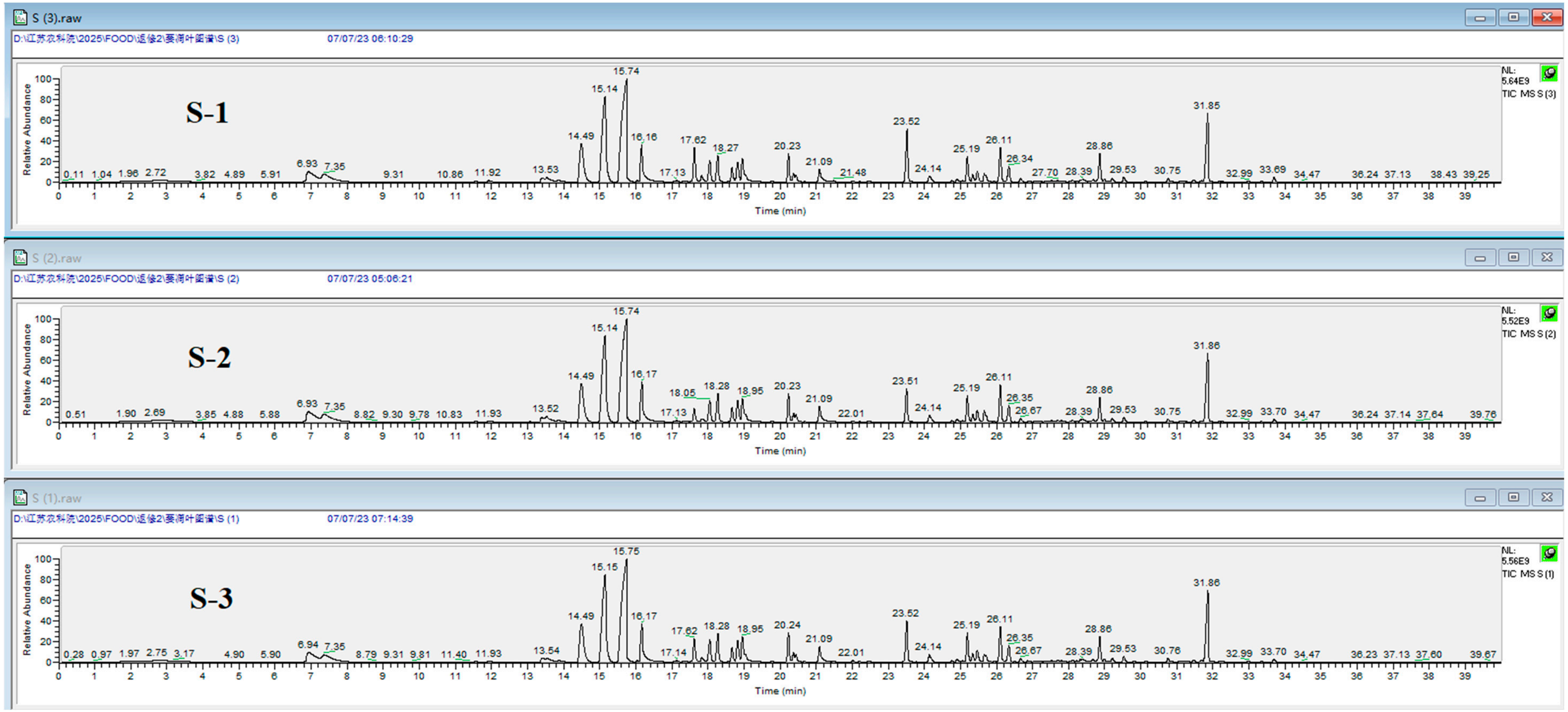

S (3).raw
07/07/23 06:10:29
S-1
NL: 5.64E9 TIC MS S(3)
S (2).raw
07/07/23 05:06:21
S-2
NL: 5.52E9 TIC MS S(2)
S (1).raw
07/07/23 07:14:39
S-3
NL: 5.56E9 TIC MS S(1)
Relative Abundance
Time (min)

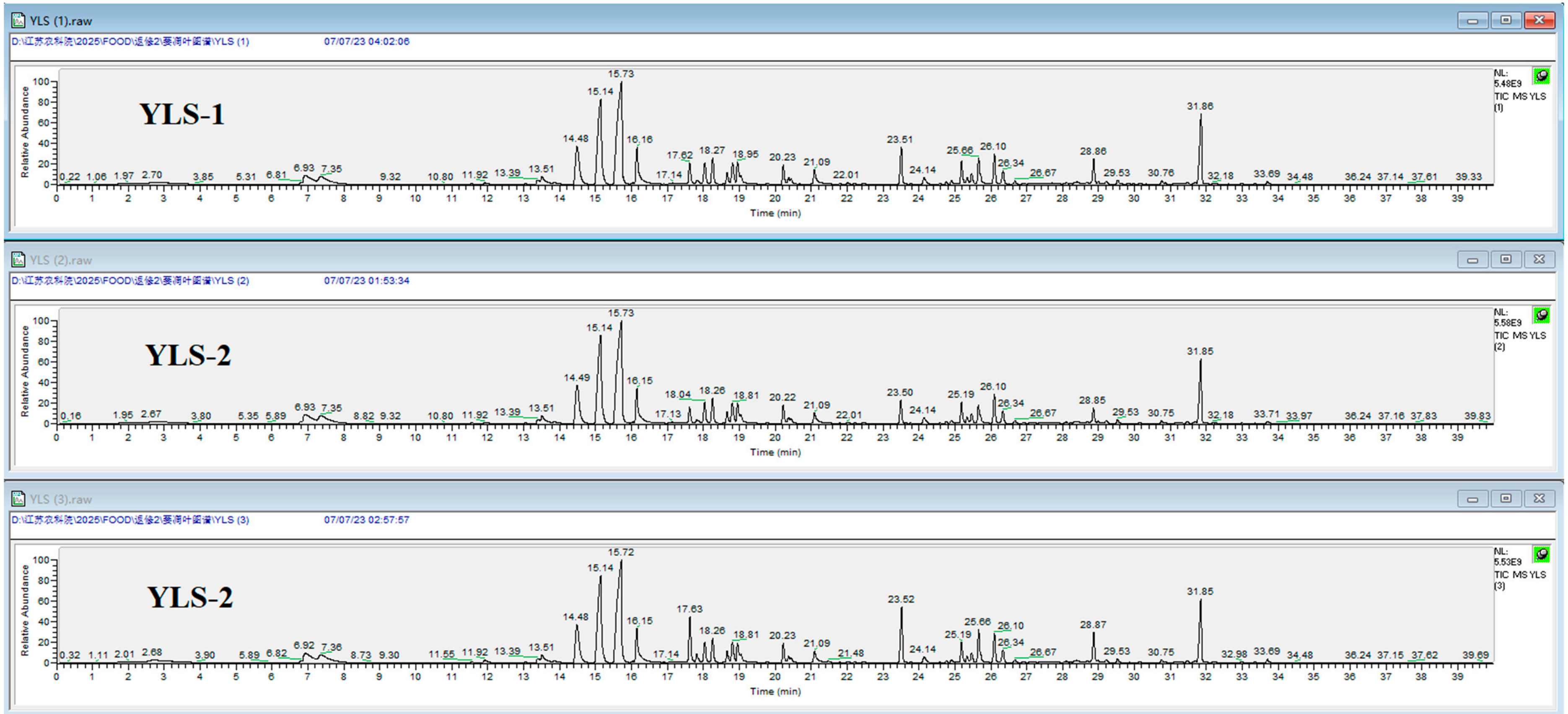
YLS (1).raw
07/07/23 04:02:06
YLS-1
NL: 5.48E9 TIC MS YLS (1)
Relative Abundance
Time (min)
0.22 1.06 1.97 2.70 3.85 5.31 6.81 6.93 7.35 9.32 10.80 11.92 13.39 13.51 14.48 15.14 15.73 16.16 17.14 17.62 18.27 18.95 20.23 21.09 22.01 23.51 24.14 25.66 26.10 26.34 26.67 28.86 29.53 30.76 31.86 32.18 33.69 34.48 36.24 37.14 37.61 39.33
YLS (2).raw
07/07/23 01:53:34
YLS-2
NL: 5.58E9 TIC MS YLS (2)
Relative Abundance
Time (min)
0.16 1.95 2.67 3.80 5.35 5.89 6.93 7.35 8.82 9.32 10.80 11.92 13.39 13.51 14.49 15.14 15.73 16.15 17.13 18.04 18.26 18.81 20.22 21.09 22.01 23.50 24.14 25.19 26.10 26.34 26.67 28.85 29.53 30.75 31.85 32.18 33.71 33.97 36.24 37.16 37.83 39.83
YLS (3).raw
07/07/23 02:57:57
YLS-2
NL: 5.53E9 TIC MS YLS (3)
Relative Abundance
Time (min)
0.32 1.11 2.01 2.68 3.90 5.89 6.82 6.92 7.36 8.73 9.30 11.55 11.92 13.39 13.51 14.48 15.14 15.72 16.15 17.14 17.63 18.26 18.81 20.23 21.09 21.48 23.52 24.14 25.19 25.66 26.10 26.34 26.67 28.87 29.53 30.75 31.85 32.98 33.69 34.48 36.24 37.15 37.62 39.69

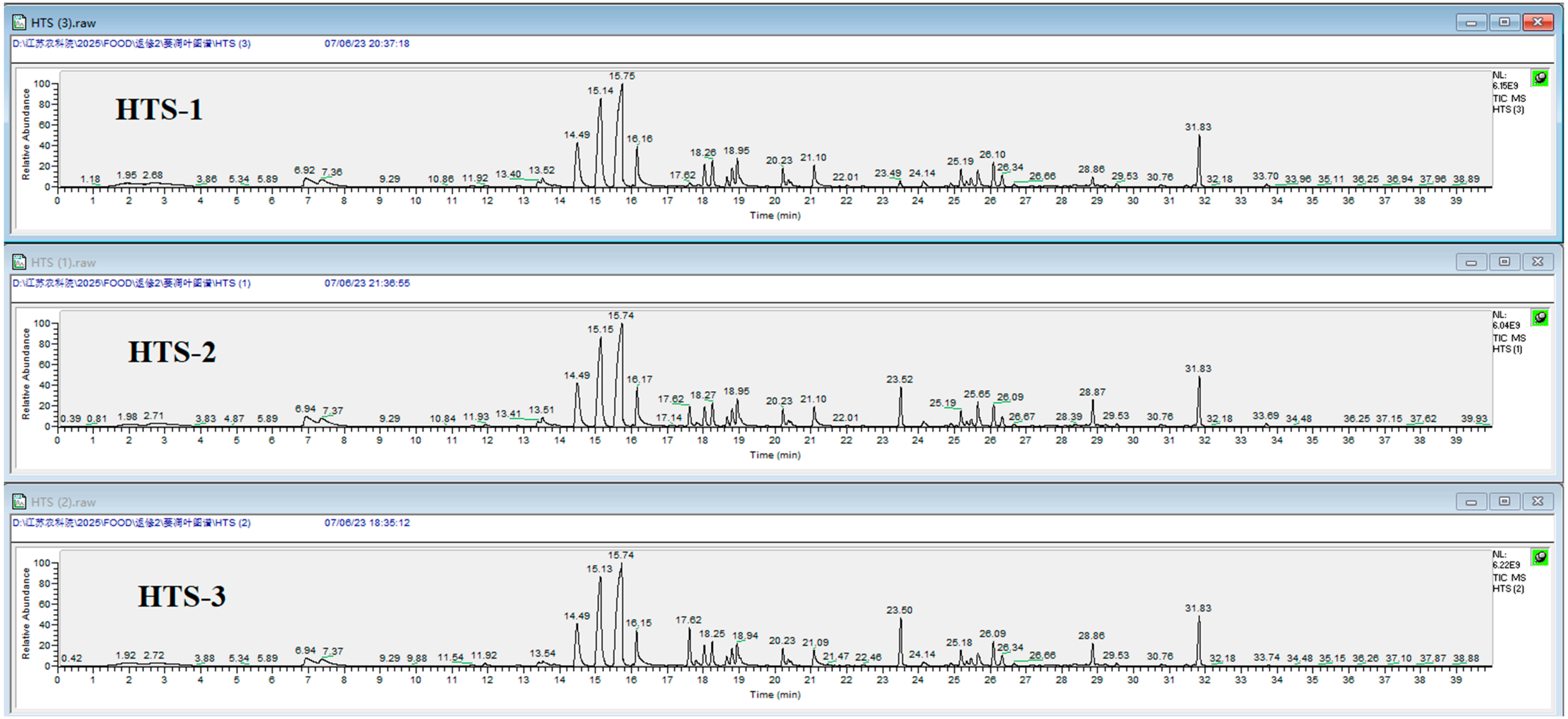
HTS (3).raw
07/06/23 20:37:18
HTS-1
NL: 6.15E9 TIC MS HTS (3)
HTS (1).raw
07/06/23 21:36:55
HTS-2
NL: 6.04E9 TIC MS HTS (1)
HTS (2).raw
07/06/23 18:35:12
HTS-3
NL: 6.22E9 TIC MS HTS (2)
Relative Abundance
Time (min)

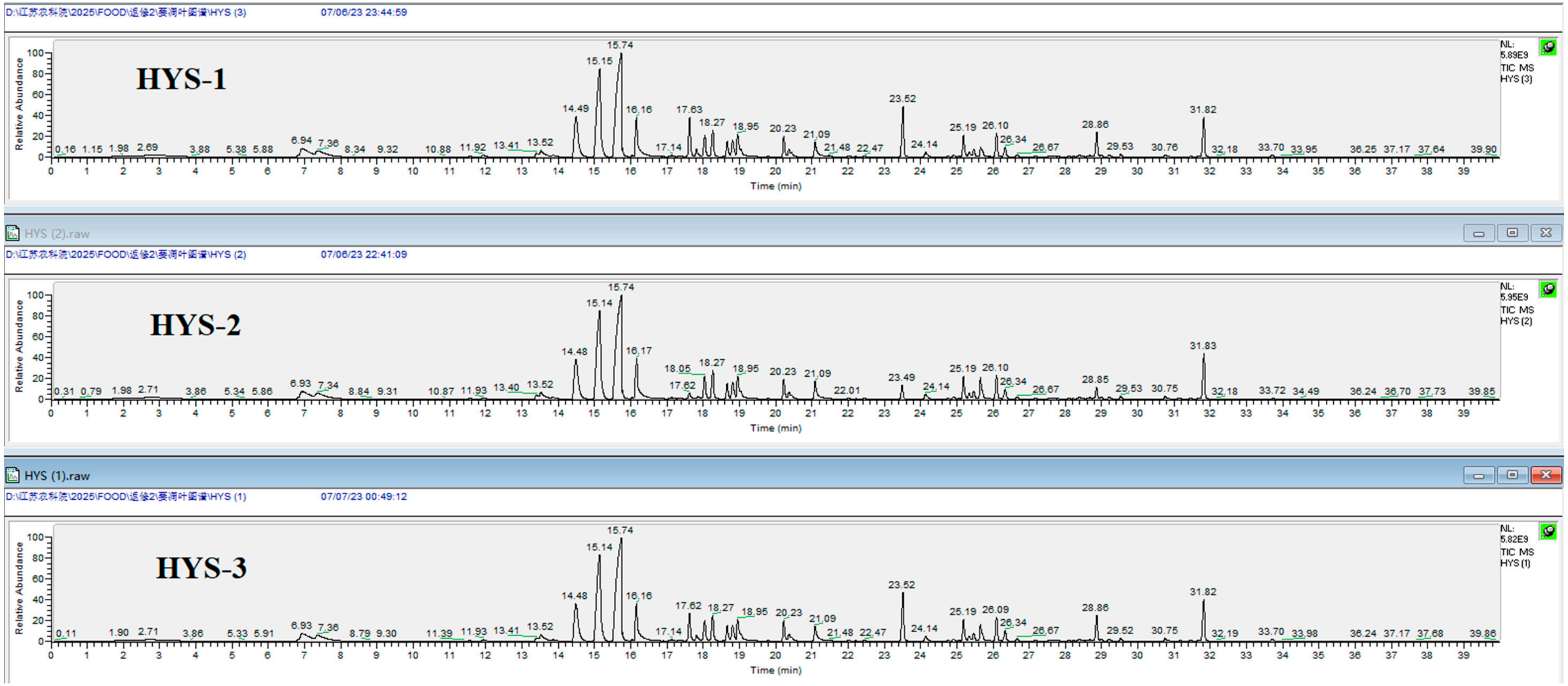

07/06/23 23:44:59
HYS-1
NL: 5.89E9 TIC MS HYS (3)
HYS (2).raw
07/06/23 22:41:09
HYS-2
NL: 5.95E9 TIC MS HYS (2)
HYS (1).raw
07/07/23 00:49:12
HYS-3
NL: 5.82E9 TIC MS HYS (1)
Relative Abundance
Time (min)
